# Hedgehog signalling is involved in acquired resistance to KRAS^G12C^ inhibitors in lung cancer cells

**DOI:** 10.1038/s41419-024-06436-9

**Published:** 2024-01-16

**Authors:** Chaeyoung Lee, Jawoon Yi, Jihwan Park, Byungyong Ahn, Young-Wook Won, JiHeung Jeon, Byung Ju Lee, Wha Ja Cho, Jeong Woo Park

**Affiliations:** 1 Department of Biological Sciences, University of Ulsan, Ulsan, Korea; 2 School of Life Sciences, Gwangju Institute of Science and Technology, Gwangju, Korea; 3 Department of Food Science and Nutrition, University of Ulsan, Ulsan, Korea; 4 Basic-Clinical Convergence Research Institute, University of Ulsan, Ulsan, Korea; 5 Department of Biomedical Engineering, University of North Texas, Texas, USA; 6 RopheLBio, B102, Seoul Forest M Tower, Seoul, Korea

**Keywords:** Non-small-cell lung cancer, Cancer therapeutic resistance

## Abstract

Although KRAS^G12C^ inhibitors have shown promising activity in lung adenocarcinomas harbouring KRAS^G12C^, acquired resistance to these therapies eventually occurs in most patients. Re-expression of KRAS is thought to be one of the main causes of acquired resistance. However, the mechanism through which cancer cells re-express KRAS is not fully understood. Here, we report that the Hedgehog signal is induced by KRAS^G12C^ inhibitors and mediates KRAS re-expression in cancer cells treated with a KRAS^G12C^ inhibitor. Further, KRAS^G12C^ inhibitors induced the formation of primary cilia and activated the Hedgehog-GLI-1 pathway. GLI-1 binds to the KRAS promoter region, enhancing KRAS promoter activity and KRAS expression. Inhibition of GLI using siRNA or the smoothened (Smo) inhibitor suppressed re-expression of KRAS in cells treated with a KRAS^G12C^ inhibitor. In addition, we demonstrate that KRAS^G12C^ inhibitors decreased Aurora kinase A (AURKA) levels in cancer cells, and inhibition of AURKA using siRNA or inhibitors led to increased expression levels of GLI-1 and KRAS even in the absence of KRAS inhibitor. Ectopic expression of AURKA attenuated the effect of KRAS^G12C^ inhibitors on the expression of GLI-1 and re-expression of KRAS. Together, these findings demonstrate the important role of AURKA, primary cilia, and Hedgehog signals in the re-expression of KRAS and therefore the induction of acquired resistance to KRAS^G12C^ inhibitors, and provide a rationale for targeting Hedgehog signalling to overcome acquired resistance to KRAS^G12C^ inhibitors.

## Introduction

The KRAS protein is a GTPase that function as a molecular switch that cycles between active GTP-bound and inactive GDP-bound states [1]. The binding of GTP to KRAS promotes the binding of effectors that trigger signal transduction pathways, including the RAF-MEK-ERK (MAPK) pathway [2, 3]. Activating mutations in the *KRAS* gene are a hallmark of cancer [4] and the KRAS^G12C^ protein mutation is one of the most common activating alterations in lung adenocarcinoma [5]. KRAS^G12C^ mutations occur in approximately 13% of lung adenocarcinomas and 3% of colorectal adenocarcinomas, and are less commonly in cancers of the uterus, pancreas, breast, bladder, cervix, and ovaries [6, 7]. A KRAS^G12C^ mutation results in an increase of GTP-bound activated KRAS [8] and activates downstream signaling pathways that allow cells to grow uncontrollably [9].

Due to the intrinsic characteristics of KRAS proteins, KRAS has been considered an undruggable target. KRAS proteins have relatively smooth surfaces, no obvious deep hydrophobic pockets to which drugs can bind [10], and high affinity for GTPs, which make it difficult to interfere with GTP binding using GTP analogs [11]. However, after discovering allosteric inhibitors that can bind cysteine 12 residues within the switch II region of the KRAS^G12C^ mutant, thereby locking the protein in its inactive GDP-bound state [12], several KRAS^G12C^ inhibitors have been identified that bind covalently to the GDP-bound form of the KRAS^G12C^ protein, preventing oncogenic signaling and causing tumor regression in preclinical models [8, 12, 13]. In early-phase clinical trials, two small-molecule KRAS^G12C^ inhibitors, adagrasib (MRTX849) and sotorasib (AMG 510), have shown promising results against non–small cell lung cancer and more modest efficacy against colorectal cancer [13, 14]. Despite the clinical benefits observed for many patients treated with KRAS^G12C^ inhibitors, most patients eventually acquire resistance to single-agent therapy. Acquired resistance to KRAS^G12C^ inhibitors can be caused by various mechanisms [15], including genetic changes such as secondary mutations in KRAS^G12C^ that interfere with the binding of covalent inhibitors to cysteine 12 residues, and other mutations related to the reactivation of the RAS-RAF-MEK-ERK signaling pathway [16, 17]. In addition to genetic changes, acquired resistance can be induced by synthesizing new KRAS^G12C^ proteins and reactivating downstream signaling [18]. Similarly, reactivation of the MAPK pathway without new mutations in KRAS or its downstream mediators has been reported in KRAS^G12C^ lung adenocarcinomas resistant to the KRAS^G12C^ inhibitor AMG 510 [19]. Further analysis has revealed that epidermal growth factor receptor or Aurora kinase A (AURKA) signals can maintain newly expressed KRAS^G12C^ proteins in an active GTP-bound form, thereby evading treatment by KRAS^G12C^ inhibitors [18]. However, another study suggests that wild-type RAS activation mediated by multiple-*receptor tyrosine kinases (*RTKs), rather than a single RTK, is responsible for acquired resistance to the KRAS^G12C^ inhibitors ARS-1620 and sotorasib in various cancer cell lines [20]. Ultimately, an improved understanding of the biological basis of drug resistance will provide more opportunities to optimize KRAS^G12C^-inhibitor regimens and new combinations.

Hedgehog (Hh) signaling plays a pivotal role in regulating a number of cell-fate and self-renewal processes during development and tissue homeostasis [21]. Vertebrate Hh signaling is transduced by the primary cilium, a cellular antenna that projects from the surface of most cells [22, 23]. The seven transmembrane domain-containing protein known as smoothened (Smo) serves as a key player in Hh signaling. In the absence of Hh ligands, the Hh receptors patched 1 (PTCH1) and PTCH2 at the cilium inhibit Smo, keeping the pathway in its off state [24]. When Hh ligands bind to their receptor PTCHs at the cilium, inhibition of Smo ceases and Smo accumulates at the cilium, where it activates the downstream pathway [25]. Upon Hh pathway activation, glioma-associated oncogene 1 (GLI-1) transcription factors are activated and regulate target gene expression by direct association with a specific consensus sequence in the promoter region of the target genes [26].

In this study, we report that, consistent with a previous report [18], mutant KRAS^G12C^ cancer cells initially responded to KRAS^G12C^ inhibitors but then rapidly re-expressed KRAS^G12C^ and re-activated its downstream extracellular signal–regulated kinase (ERK). These changes are associated with upregulation of the Hh signal, cilia frequency, and cilia length. Inhibition of the Hh signaling pathway blocks re-expression of KRAS^G12C^, reactivation of ERK, and resumption of cell proliferation after treatment with a KRAS^G12C^ inhibitor. Our results suggest that upregulation of the Hh pathway plays a critical role in inducing acquired resistance of mutant KRAS^G12C^ cancer cells against KRAS^G12C^ inhibitors, providing a rationale for targeting the Hh pathway as a strategy to overcome acquired resistance to KRAS^G12C^ inhibitors.

## Materials and methods

### Cell culture and reagents

Human non–small cell lung carcinoma H358 (CRL-5807, KCLB 25807) and H23 (CRL-5800, KCLB 90023) cell lines harboring the KRAS^G12C^ mutation were purchased from the Korean Cell Line Bank (KCLB, Seoul, Korea). Cells were maintained in RPMI-1640 medium supplemented with 10% fetal bovine serum, penicillin, streptomycin, and 2 mM L-glutamine. The KRAS^G12C^ inhibitors ARS-1620 (S8707) and AMG 510 (S8830), Smo inhibitor sonidegib (S2151), and AURKA inhibitor Tozasertib (S1048) were purchased from Selleck Chemicals (Selleck Chemicals LLC, Houston, TX, USA) and administered at 10 μM, or as otherwise specified, in culture.

### RAS-GTP pull-down assay

Determination of GTP-bound KRAS levels was performed using the Active Ras Pull-Down and Detection Kit (Thermo Fisher Scientific, 16117) according to the manufacturer’s instruction. Briefly, whole-cell lysates were incubated with recombinant glutathione *S*-transferase (GST)-tagged Raf1 RBD protein, followed by Glutathione-agarose beads, to isolate the bound KRAS-GTP. The samples were then subjected to western blotting with an anti-KRAS antibody (Sigma, WH0003845M1). Total KRAS was determined by blot analyses with an anti-KRAS antibody (Sigma, WH0003845M1) using the whole-cell lysate.

### SDS-PAGE and immunoblotting

Cells were lysed in a RIPA lysis and extraction buffer (Thermo Fisher Scientific) with protease and phosphatase inhibitors (Thermo Fisher Scientific) on ice. Samples were separated by sodium dodecyl sulfate–polyacrylamide gel electrophoresis (SDS-PAGE) followed by transfer to nitrocellulose membranes (GE, 10600001). Membranes were probed with primary antibodies against mouse anti-ERK (Cell Signaling, 9107), rabbit anti-phospho-Erk (Cell Signaling, 9101), rabbit anti-IFT88 (Proteintech, 13967-1-AP), mouse anti-ARL6 (Proteintech, 12676-1-AP), rabbit anti-GLI-1 (Cell Signaling, 2553), rabbit anti-AURKA (Cell Signaling, 4718), and mouse anti-β-actin (Sigma, A5441). Immunoreactivity bands were detected using a Pierce electrochemiluminescence western blotting substrate (Thermo Fisher Scientific).

### Quantitative and semiquantitative real-time PCR

Total RNA from cells was extracted using a TRIzol reagent (Invitrogen). Synthesis of cDNA used 2 μg of total RNA, oligo-dT, and Superscript II reverse transcriptase (Invitrogen). Templates for quantitative real-time polymerase chain reaction (qRT-PCR) amplification of gene specific primers are listed in Table 1. All qRT-PCR analyses included real-time monitoring of the increase in fluorescence of SYBR Green dye (QIAGEN, Hilden, Germany) using a StepOne real-time PCR system (Applied Biosystems). Semiquantitative RT-PCR was performed using a Taq polymerase (Solgent, Daejeon, Korea).

**Table 1:** PCR primers used in this study.

Primers			Sequences (5′-3′)
Gene expression analysis	ARL6	F	GGCTTCAAGATCAGATCCAGACT
		R	AAGGTCAGAGTCCATAAATGCAAG
	β-actin	F	ATCGTGCGTGACATTAAGGAGAAG
		R	AGGAAGGAAGGCTGCAAG
	AURKA	F	GCAACCAGTGTACCTCATCCTG
		R	AAGTCTTCCAAAGCCCACTGCC
	GAPDH	F	AATCCCATCACCATCTTCCAG
		R	AAATGAGCCCCAGCCTTC
	GLI1	F	ACAGCCAGTGTCCTCGACTT
		R	ATAGGGGCCTGACTGGAGAT
	IFT88	F	GCCGAAGCACTTAACACTTAT
		R	GTCTAATGCCATTCGGTAGAA
	KRAS	F	TCGACACAGCAGGTCAAGAG
		R	CAAAGAAAGCCCTCCCCAGT
	PTCH1	F	CTCCTTTGCGGTGGACAA
		R	CCTCAGCCTTATTCAGCATTTC
ChIP analysis	KRAS-P1	F	CTAGGAGGGGGAGACTGGAA
		R	AGCGCCTGTACCTGATAGGA
	KRAS-P2	F	TCCTATCAGGTACAGGCGCT
		R	CTCCACAGAGAAGCTGCGAA
	KRAS-P3	F	TAAGTCCCCGAAGTCGCCTC
		R	CTGCCTAGCCGCAAGGCTGT
	KRAS-P4	F	ACAGCCTTGCGGCTAGGCAG
		R	AAATCGAGCTCCGAGCACACCG
	KRAS-P5	F	GGAAAGGATGACAGTTGATGTAAAG
		R	TCCGAGACTTTCAGTTCCATTC

### Cell cycle analysis

The effect of the KRAS^G12C^ inhibitor ARS-1620 on cell cycle distribution was examined by propidium iodide staining followed by FACS Scan Flow cytometry analysis. Briefly, H358 cells at 50% confluency were treated with 10 μM ARS-1620 for the indicated times. Cells were collected and fixed in ice-cold 70% ethanol at 4 °C for 24 h. After washing with phosphate-buffered solution (PBS), cells were stained by incubating them with a freshly made PBS solution containing 20 μg/mL propidium iodide (Sigma-Aldrich) and 0.2 mg/mL ribonuclease A (Sigma-Aldrich) at 37 °C for 30 min in the dark. Cell cycle distribution in the prepared cells was estimated by measuring the cell’s DNA content according to the standard procedures using FACS Canto II (Beckton Dickinson).

### Immunostaining and quantification of ciliated cells

Cells were treated with a combination of 10 μM ARS-1620 and 10 μM Sonidegib for 72 h and fixed with 4% paraformaldehyde and 0.3% (v/v) Triton X-100 in PBS for 10 min. After blocking the cells with 5% bovine serum albumin (BSA) in PBS, the cells were incubated overnight at 4 °C with primary antibodies against acetylated α-tubulin (T7451, Sigma-Aldrich) and a small GTPase Arl13B enriched in primary cilia [27] (17711-1-AP, Proteintech) in 1% BSA. After washing, the cells were incubated with Alexa Fluor-conjugated secondary antibodies (Life Technologies) at room temperature for 1 h. Nuclei were detected with 1 μg/mL 4′,6-diamidino-2-phenylindole (Sigma-Aldrich). The stained cells were examined using an Olympus 1000/1200 laser-scanning confocal system. Cilia were counted in approximately 150 cells under each experimental condition. The percentage of ciliated cells was calculated as (total number of cilia)/(total number of DAPI-labeled nucleus at each image) × 100. Cilia lengths were measured using the Free-hand Line Selection Tool of Cell Sense Standards software (Olympus Europa Holding GmbH, Hamburg, Germany) and average cilium lengths were calculated. Data analysis was performed in GraphPad Prism 8 (GraphPad Software, San Diego, CA).

### RNA preparation and RNA-seq

We performed RNA-seq analysis on total RNA samples (RIN > 8.5) collected from H358 cells at 0, 12, and 48 h after treatment with 10 μM ARS-1620 or 10 μM Sonidegib. Residual DNA from each sample was removed using the RNeasy MinElute Cleanup Kit (Qiagen, Hilden, Germany). Libraries of cDNA were prepared with 1.0 μg of total RNA using the TrueSeq RNA Library Preparation Kit (Illumina, San Diego, CA, USA) following the manufacturer’s recommendations, followed by paired-end sequencing (2 × 100 bp) using the HiSeq1500 platform (Illumina, San Diego, CA, USA). Amplification of cDNA was carried out according to the RNA-seq protocol provided by Illumina and sequenced using an Illumina HiSeq 2500 system to obtain 150-bp paired-end reads. The sequencing depth for each sample was > 20 million reads. RNA-seq reads were mapped to the human genome GRCh38/hg38 using STAR 2.7.9a [28]. The featureCounts function of the Rsubread package was used to generate count tables. The DESeq2 package [29] was used to generate a DEG list object from feature counts. Genes with a false discovery rate (adjusted *P* value < 0.05 and an absolute log2 fold change > 1.5) were selected as DEGs. Normalized read counts were used for hierarchical clustering by hclust in R. The count table generated by featureCounts was subjected to further GSEA (Java, GSEA Desktop Application version 3.0; http://software.broadinstitute.org/gsea/downloads.jsp). Positively and negatively enriched pathways with a cut-off false discovery rate of *P* < 0.25 were considered significant pathways. The top 20 positively or negatively enriched pathways for each group were then depicted in bar plots based on normalized enrichment scores.

### Clonogenic assay

Cells were seeded into six-well plates (3.5 × 10^5^ cells per well) overnight and treated with 10 μM ARS-1620 and 10 μM sonidegib. The medium with drugs was replaced every 3 days. On the indicated day, the media were aspirated, and cells were washed with PBS. A 0.5% crystal violet (Sigma, HT90132), 20% methanol solution was added to the cells. Cells were incubated with rocking for 30 min, after which the crystal violet was discarded and plates were left to dry overnight.

### Chromatin immunoprecipitation assay

Chromatin immunoprecipitation assays were carried out using the EZ-Magna ChIPTM G Kit (Millipore 17-611) according to the manufacturer’s instruction. H358 cells were treated with a combination of 10 μM ARS-1620 and 10 μM sonidegib for 48 h. Nuclei were isolated from the cells and sonicated to shear the DNA, which was distributed around 0.2 kb to 1 kb on 1% agarose gel. Chromatin was immunoprecipitated with anti-GLI-1 (Novus, NB 600-600), anti-histone H3 (Cell Signaling, 4620) or an isotype control (Cell Signaling, 2729). The complexes were collected on Protein G Magnetic beads and subsequently extracted from the beads. Bound DNA was purified and amplified by qRT-PCR with primers that amplify 204-bp (−1262 to −1059), 618-bp (-1078 to -460), 260-bp (-594 to -335), 354-bp (-354 to -1), and 117-bp (−1205 to −1089) regions (Table 1 and Fig. S4) of the KRAS promoter.

### Plasmids, small interfering RNAs, transfections, and luciferase assay

The pAuroraA GFP-AURKA-GFP (deposited by Marc Tramier, Institute Genetics & Development of Rennes, Rennes, France) was purchased from Addgene (#157765, addgene.org). The pCMV6-Entry GLI1 was purchased from Origene (RC201110, Rockville, MD, USA). The KRAS promoter reporter pEZX-PG04-KRAS was purchased from GeneCopoeia (HPRM45839-PG04, Rockville, MD, USA). Cells were transfected with plasmid vectors using TurboFect (Thermo Fisher Scientific). Small interfering RNAs (siRNAs) against human AURKA (AURKA-siRNA, ID s195), human GLI-1 (siGLI-1 #1) (GLI-1-siRNA, ID 107671), and control-scrambled siRNA (scRNA, AM4611) were purchased from Thermo Fisher Scientific. The second siRNA against human GLI-1 (siGLI-1 #2) was synthesized by Integrated DNA Technologies (Coralville, IA) and the sequences were as follows: sense 5’-GCGAAAACAUGUCAAGACAGUGCAT-3’, antisense 5’-AUGCACUGUCUUGACAUGUUUUCGCAG-3’. Cells were transfected with siRNA using Lipofectamine RNAiMAX (Invitrogen). The expression levels of mRNA were analyzed by qRT-PCR. Luciferase activity was measured using a Secrete-Pair Dual Luminescence Assay kit (GeneCopoeia), according to the manufacturer’s instructions, and a SpectraMax L Microplate (Molecular Devices, Sunnyvale, CA, USA). All luciferase assays reported in this study are based on at least three independent experiments, each consisting of three wells per transfection.

### Statistical analysis

Differences in the expression of genes were evaluated by Student’s t-tests or one-way analysis of variance. A *P* value of less than 0.05 was considered statistically significant.

## Results

### KRAS^G12C^-mutant cancer cells initially respond to KRAS^G12C^ inhibitors but rapidly re-express KRAS^G12C^ and reactivate ERK

**Fig. 1:**
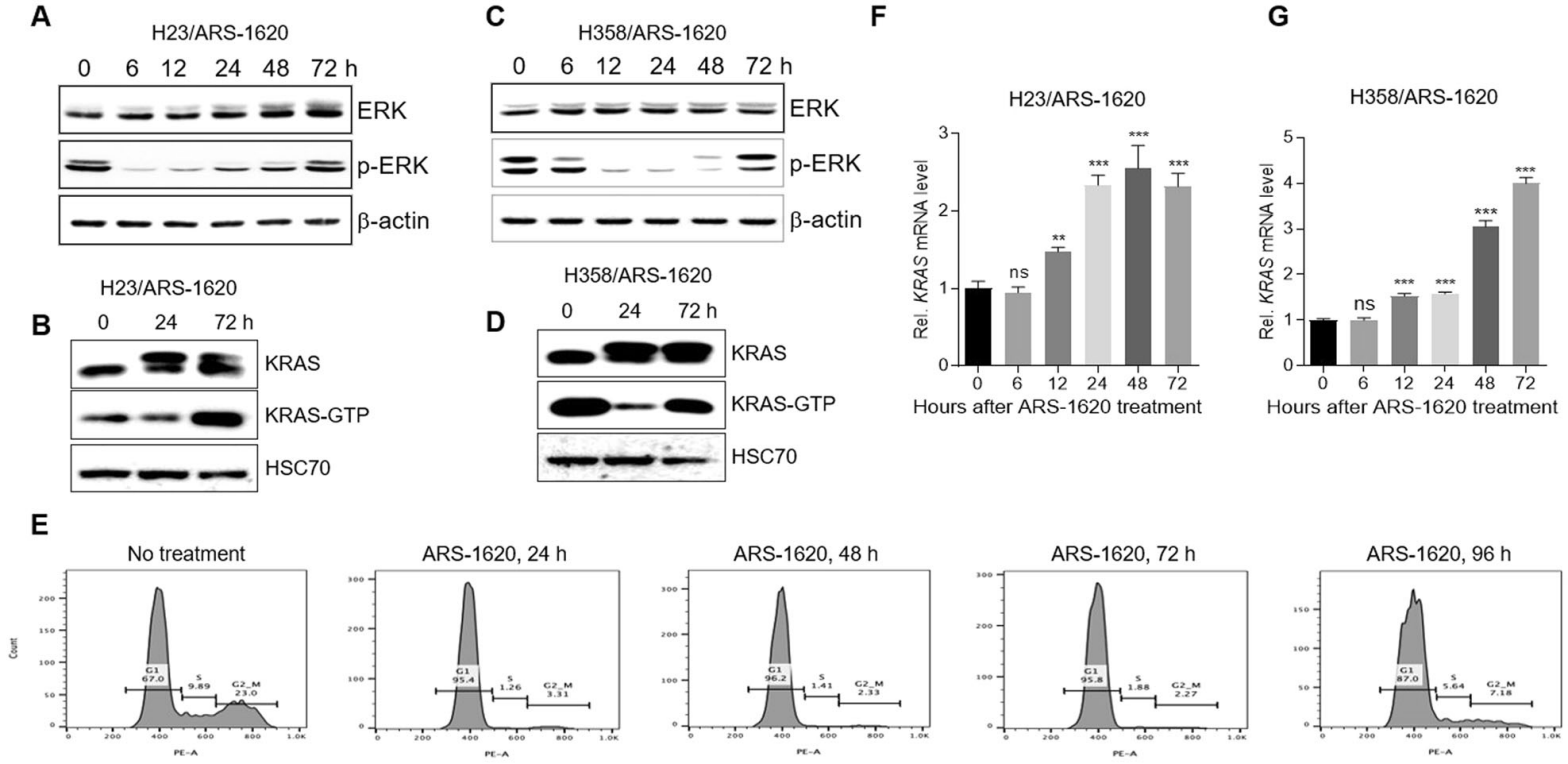
The KRAS^G12C^ inhibitor ARS-1620 induces re-expression of KRAS and reactivation of ERK signal in lung cancer cells. **A**, **B** and **F** H23 and **C**, **D** and **G** H358 lung cancer cells were treated with 10 μM ARS-1620 for the indicated times. Time-dependent changes in ERK phosphorylation and accumulation of KRAS-GTP in H23 (**A**, **B**) and H358 cells (**C**, **D**) were determined by western blot analysis. Time-dependent changes in *KRAS* mRNA levels in H23 (**F**) and H358 cells (**G**) were determined by qRT-PCR. Fold-change in expression level was calculated relative to the values at time 0 for each cell. The graphs are mean ± standard deviation (*n* = 3 independent experiments) (one-way ANOVA, **P < 0.01; ***P < 0.001). **E** Representative plots of flow cytometry using propidium iodide staining for cell cycle analysis of H358 cells. Graphs represent the mean ± standard deviation (*n* = 3 independent experiments).

Acquired resistance against KRAS^G12C^ inhibitors reportedly can be induced by synthesizing new KRAS^G12C^ and reactivating its downstream signaling [18, 19]. We first tested the time-dependent effect of the KRAS^G12C^ inhibitor ARS-1620 on the expression of KRAS^G12C^, ERK phosphorylation, accumulation of active KRAS (KRAS-GTP), and the cell cycle of KRAS^G12C^-mutant lung cancer cells. Consistent with a previous report [18], ARS-1620 initially suppressed ERK phosphorylation and accumulation of KRAS-GTP in both H23 and H358 KRAS^G12C^-mutant lung cancer cells (Fig. 1A–D and Supplemental Material 1). ARS-1620 also initially arrested the cell cycle at the G1 phase (Fig. 1E). However, both H23 and H358 cells began to express KRAS within 12 h of ARS-1620 treatment (Fig. 1F, G), reaccumulate KRAS-GTP, and reactivate ERK over time (Fig. 1A–D and Supplemental Material 1). In addition, by 72 h after ARS-1620 treatment, populations of cells at the S and G2/M phases increased (Fig. 1E). A similar pattern of re-expression of KRAS and reactivation of ERK was observed in both H23 and H358 cells after treatment with another KRAS^G12C^ inhibitor, AMG 510 (Fig. S1 and Supplemental Material 2). These results suggest that KRAS^G12C^-mutant lung cancer cells eventually avoid the inhibitory effect of KRAS^G12C^ inhibitors through rapid re-expression of KRAS and reactivation of ERK.

### RNA-seq analysis reveals the induction of Hh pathway in KRAS^G12C^-mutant cancer cells after KRAS^G12C^ inhibitor treatment

**Fig. 2:**
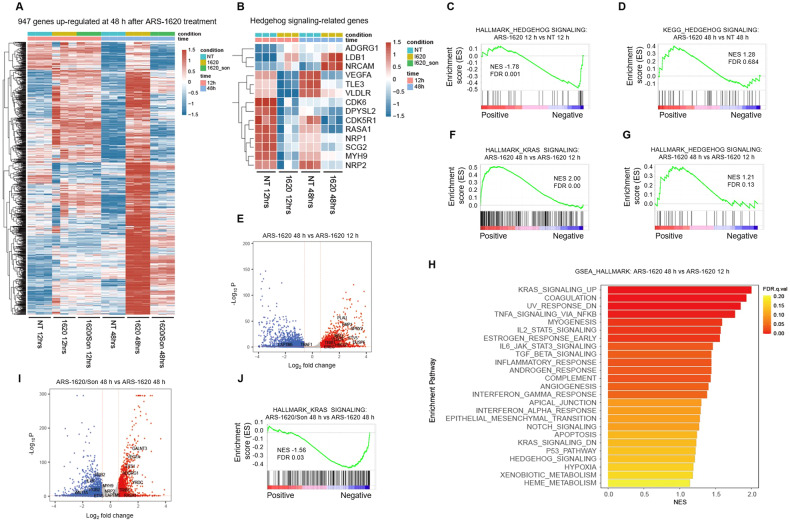
RNA-seq analysis reveals induced Hedgehog signals in lung cancer cells at 48 h after ARS-1620 treatment. H358 cells were treated with combination of 10 μM ARS-1620 and 10 μM sonidegib for 12 h or 48 h and their transcriptome profiles were analyzed by RNA-seq. **A** Heat map of 947 genes upregulated at 48 h after treatment with ARS-1620 in H358 cells versus non-treated cells (*n* = 3 independent experiments). **B** Heat map of 14 Hedgehog signaling–related genes. **C**, **D** Gene set enrichment plots of Hedgehog signaling was negatively enriched in cells at 12 h after ARS-1620 treatment (**C**) and positively enriched in cells at 48 h after ARS-1620 treatment versus non-treated cells (**D**). **E** Volcano plots of differentially expressed genes (DEGs) in cells treated with ARS-1620 for 48 h compared with cells treated with ARS-1620 for 12 h. The y-axis corresponds to the significance level represented by the −log_10_ *P* value, and the x-axis displays the log_2_ (FC) value. Red dots represent significant (adj. *P* < 0.05 and |log2FC | ≥ 1.5) DEGs. Dotted horizontal line indicates *P* = 0.05, and the dotted vertical line indicates a mean |log2FC| of 1.5. **F**–**J** Gene set enrichment plots of **F** KRAS signaling and **G** Hedgehog signaling positively were enriched in cells treated with ARS-1620 for 48 h versus cells treated with ARS-1620 for 12 h. **H** Bar plot of enriched GSEA pathways in cells treated with ARS-1620 for 48 h versus cells treated with ARS-1620 for 12 h. **I** Enrichment plot of KRAS signaling negatively enriched in cells treated with ARS-1620 and sonidegib for 48 h versus cells treated with ARS-1620 for 48 h. **J** Bar plot of enriched GSEA pathways in cells treated with ARS-1620 and sonidegib for 48 h versus cells treated with ARS-1620 for 48 h.

To examine the effect of KRAS^G12C^ inhibitors on gene expression profiles in KRAS^G12C^-mutant cancer cells, we conducted RNA-seq analysis using RNA isolated from H358 cells at 12 h and 48 h after treatment with the KRAS^G12C^ inhibitor ARS-1620. A total of 2,410 differentially expressed genes (DEGs) (adjusted *P* value < 0.05) with an absolute log2FC > 1.5 was detected using DESeq2 [29] in H358 cells treated with ARS-1620 for 12 h and 48 h compared with non-treated cells (Table S1 and S2) as shown in a heat map (Fig. 2A) and volcano plots (Fig. S2A and S2B). Two-way unsupervised hierarchical clustering of the union of DEGs showed a clear separation of ARS-1620–treated cells from non-treated cells (Fig. S2C). In addition, H358 cells treated with ARS-1620 for 12 h were clearly separated from those treated with ARS-1620 for 48 h (Fig. S2C). Hallmark and Kyoto Encyclopedia of Genes and Genomes (KEGG) gene set enrichment analysis (GSEA) showed that KRAS signaling (Fig. S2D-S2E), the cell cycle (Fig. S2F), and the G2/M checkpoint (Fig. S2G) were negatively enriched in H358 cells treated with ARS-1620 for 12 h and 48 h. While Hh signaling was negatively enriched at 12 h after ARS-1620 treatment by Hallmark GSEA, it was positively enriched at 48 h after ARS-1620 treatment by KEGG GSEA (Fig. 2B–D).

A comparison of H358 cells treated with ARS-1620 for 48 h against H358 cells treated with ARS-1620 for 12 h identified 1,287 DEGs, with 567 upregulated and 720 downregulated DEGs (adjusted *P* value < 0.05 and an absolute log2FC > 1.5) (Table S3), as shown in a volcano plot (Fig. 2E). Hallmark GSEA identified KRAS signaling as the most significantly positively enriched gene set in H358 cells treated with ARS-1620 for 48 h compared with those treated with ARS-1620 for 12 h (Fig. 2F, G), indicating that KRAS signaling was upregulated for 48 h after treatment with ARS-1620. In addition, consistent with Fig. 2B–D, Hh signaling was positively enriched by Hallmark GSEA in H358 cells treated with ARS-1620 for 48 h compared with those treated with ARS-1620 for 12 h (Fig. 2H). Collectively, the results of the RNA-seq analysis suggest that, as indicated in Fig. 1D, E, KRAS signaling is initially suppressed but rapidly re-activated in cells treated with KRAS^G12C^ inhibitors. Considering the role of Hh signaling in the expression of genes involved in cell proliferation [30, 31], it is possible that upregulation of Hh signaling plays a role in upregulating KRAS signaling at 48 h after ARS-1620 treatment. To confirm this hypothesis, RNA-seq analysis was conducted using RNA from H358 cells co-treated with ARS-1620 and the Hh signal inhibitor sonidegib. Hallmark GSEA showed that KRAS signaling was negatively enriched in H358 cells co-treated with ARS-1620 and sonidegib for 48 h compared with those treated with ARS-1620 for 48 h (Fig. 2I, J). These results suggest that the Hh signal is at least partly responsible for upregulation of KRAS signals at 48 h after ARS-1620 treatment.

### KRAS^G12C^ inhibitors enhance the Hh signal and primary cilia in cancer cells

**Fig. 3:**
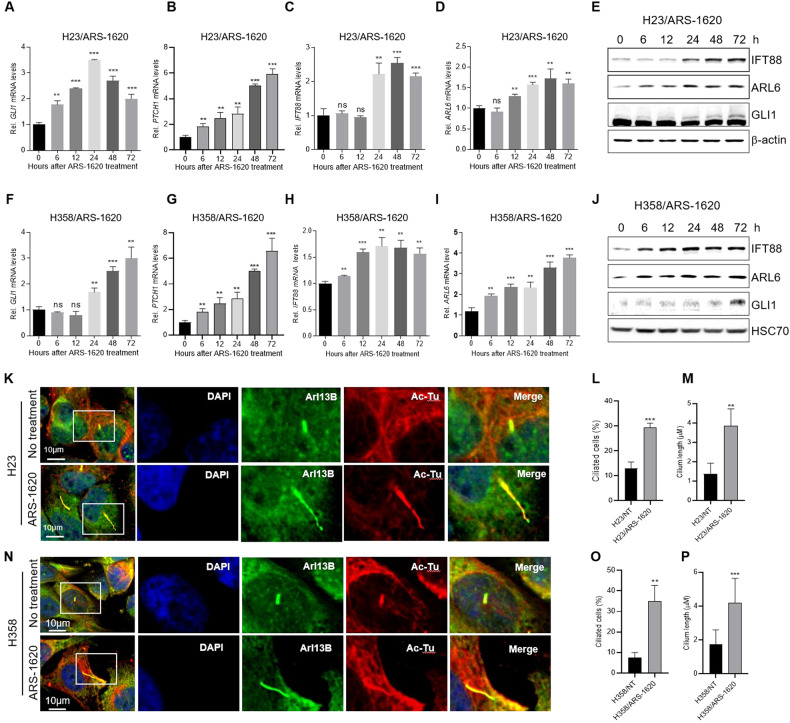
The KRAS^G12C^ inhibitor ARS-1620 induces primary cilia formation and Hedgehog signaling in lung cancer cells. **A**–**J** The KRAS^G12C^ inhibitor ARS-1620 enhances Hedgehog signaling in lung cancer cells. **A**–**E** H23 and **F**–**J** H358 cells were treated with 10 μM ARS-1620 for the indicated times. Time-dependent changes in *GLI-1* (**A**, **F)**, *PTCH1* (**B**, **G)**, *IFT88* (**C**, **H)**, and *ARL6* (**D**, **I)** mRNA levels were determined by qRT-PCR amplification. Fold change in expression levels was calculated relative to the values at time 0 for each cell. The graphs are mean ± standard deviation of three independent experiments (one-way ANOVA, **P < 0.01; ***P < 0.001). Time-dependent changes in ERK phosphorylation in H23 (**E**) and H358 cells (**J**) were determined by western blot analysis. **K**–**P** The KRAS^G12C^ inhibitor ARS-1620 induces primary cilia formation in lung cancer cells. **K**–**M** H23 and **N**–**P** H358 cells were treated with 10 μM ARS-1620 for 72 h and then stained for acetylated tubulin (Ac-Tu, red), Arl13B (green), and DAPI (blue). **K**, **N** Representative confocal microscopy images of H23 (**K**) and H358 (**N**) cells. **L**, **O**, **M**, **P** Percentages of ciliated H23 (**L**) and H358 cells (**O**) and the average length of cilia of H23 (**M**) and H358 cells (**P**) presented as the mean ± standard deviation (*n* = 150 pooled from three independent experiments). Student’s t-test, *P < 0.05; **P < 0.01; ***P < 0.001.

When an Hh signal is upregulated, GLI transcription factors are activated and induce the transcription of their target genes, such as GLI-1 and PTCH1 [32, 33]. Upregulation of Hh signals in GSEA of KRAS^G12C^ inhibitor–treated cells (Fig. 2F–H) prompted us to test whether KRAS^G12C^ inhibitors increase expression of genes involved in the Hh pathway in cancer cells. Treatment of the KRAS^G12C^ inhibitor ARS-1620 increased the expression of GLI-1, PTCH1, IFT88, and ARL6 in H23 (Fig. 3A–E and Supplemental Material 3) and H358 cells (Fig. 3F–J and Supplemental Material 3). Another KRAS^G12C^ inhibitor, AMG 510, also enhanced the expression of GLI-1, PTCH1, and IFT88 in H23 and H358 cells (Fig. S3). Primary cilia, which are microtubule-based organelles, are required for transduction of Hh signals in vertebrates [21]. We therefore investigated whether KRAS^G12C^ inhibitors affect primary cilia formation by staining cells for the primary cilia membrane Arl13b and acetylated α-tubulin (Ac-Tu). Consistent with Hh signals, immunofluorescent labeling of primary cilia revealed that the proportion of cells exhibiting a cilium and the primary cilia length increased in both H23 (Fig. 3K–M) and H358 cells (Fig. 3N–P) after ARS-1620 treatment.

### Downregulation of AURKA mediates the induction of Hh signaling and re-expression of KRAS after treatment with a KRAS^G12C^ inhibitor

**Fig. 4:**
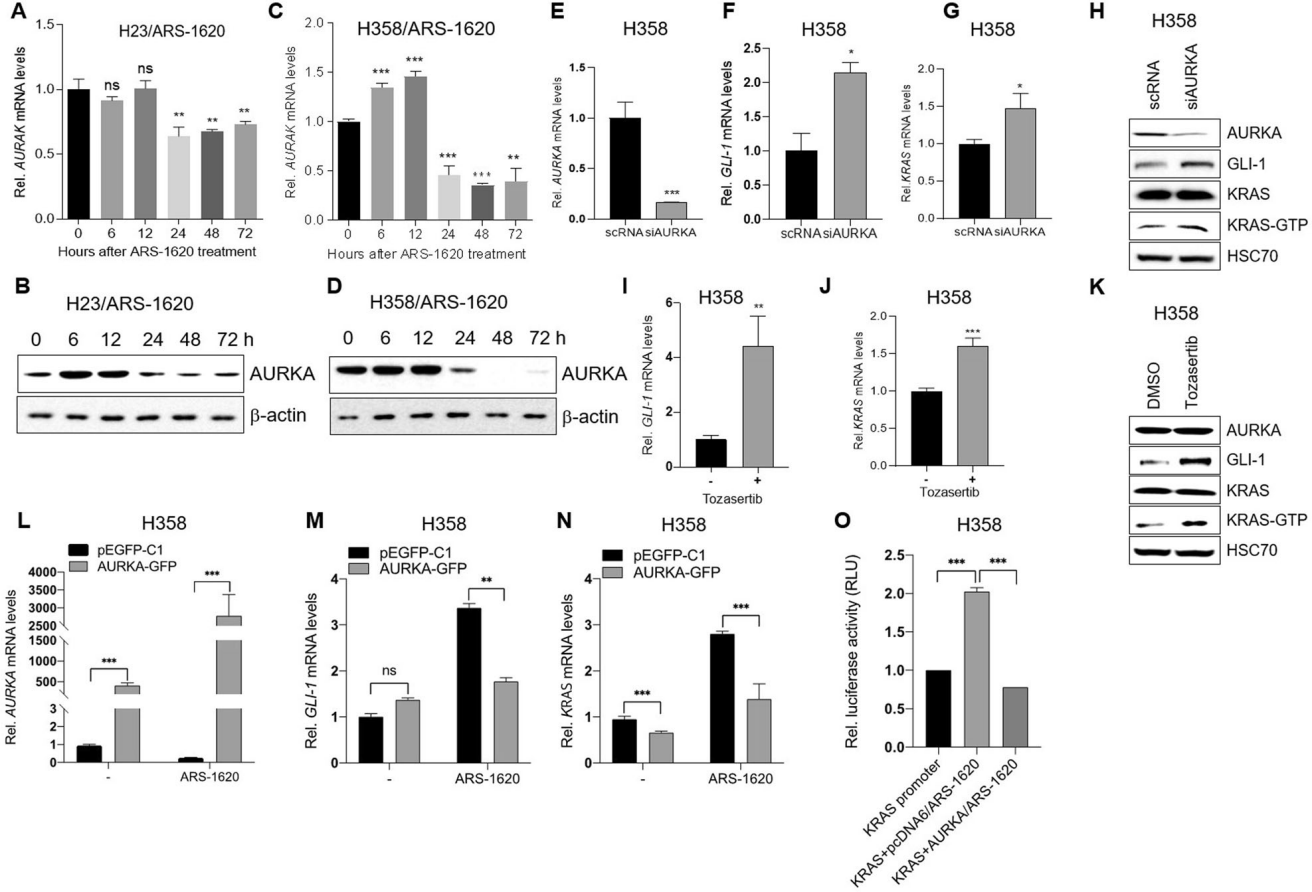
Downregulation of AURKA by ARS-1620 mediates the induction of Hedgehog signaling and re-expression of KRAS. **A**–**D** ARS-1620 treatment downregulates the expression of AURKA in lung cancer cells. H23 (**A**, **B)** and H358 (**C**, **D)** cells were treated with 10 μM ARS-1620 for the indicated times. Time-dependent changes in mRNA (**A**, **C)** and protein levels of AURKA (**B**, **D)** were determined by qRT-PCR and western blot analysis, respectively. Fold change in expression levels was calculated relative to the values at time 0 for each cell. The graphs are mean ± standard deviation of three independent experiments (one-way ANOVA, **P < 0.01; ***P < 0.001). **E**–**K** Inhibition of AURKA using siRNA (**E–H**) or an inhibitor (Tozasertib) (**I–K**) induced Hedgehog signaling and accumulation of KRAS-GTP. H358 cells were transfected with control scRNA or siRNA against AURKA (siAURKA) or treated with a 10 μM inhibitor. Expression levels of AURKA (**E**, **H**, and **K**), GLI-1 (**F**, **H**, **I**, and **K**), and KRAS (**G**, **H**, **J**, and **K**) were determined by qRT-PCR amplification and Western blot analysis. **L**–**N** Ectopic expression of AURKA attenuates the induction of Hedgehog signals and re-expression of KRAS in ARS-1620-treated cells. H358 cells were transfected with p-Aurora A GFP-AURKA-GFP expression vector or empty vector as a control, followed by treatment with 10 μM ARS-1620 for the indicated times. **L** Expression of AURKA was confirmed by qRT-PCR amplification, as were time-dependent changes in **M** GLI-1, and **N** KRAS mRNAs. Fold-change in expression levels was calculated relative to the values at time 0 for each cell. The graphs are mean ± standard deviation of three independent experiments (one-way ANOVA, **P < 0.01; ***P < 0.001). **O** Ectopic expression of AURKA blocks ARS-1620–mediated activation of KRAS promoter. H358 cells were co-transfected with the luciferase reporter vector pEZX-PG04.1/KRAS promoter and p-Aurora A GFP-AURKA-GFP expression vector or empty vector served as a control, followed by treatment with 10 μM ARS-1620 for 48 h. The same volume of dimethylsulfoxide was added to the cells as controls. The fold change in luciferase activity was calculated relative to that of empty vector-transfected dimethylsulfoxide control. The graphs are the mean ± standard deviation of three independent experiments (one-way ANOVA, ***P < 0.001).

AURKA plays a key role in primary cilia disassembly [34]. AURKA is activated in the G2/M phase, and most AURKA proteins undergo degradation after mitosis [35]. The cell cycle of cancer cells treated with KRAS^G12C^ inhibitors has been reported to be arrested at G0/G1 [18]. When we analyzed the expression level of AURKA in cancer cells, we found that the KRAS^G12C^ inhibitor ARS-1620 decreased both mRNA and protein levels of AURKA in H23 (Fig. 4A, B and Supplemental Material 4) and H358 cells (Fig. 4C, D and Supplemental Material 4) at 24 h post-treatment but increased them slightly at 72 h post-treatment. These results suggest that reduced levels of AURKA at 24 h post-treatment may play a role in induction of Hh signals and therefore re-expression of KRAS in cancer cells treated with a KRAS^G12C^ inhibitor. Inhibition of AURKA using siRNA (Fig. 4E–H or the inhibitor Tozasertib (Fig. 4I–K) consistently increased the expression of GLI-1 (Fig. 4F, H, I, and K and Supplemental Material 4) and KRAS (Fig. 4G, H, J, K, and Supplemental Material 4) in the absence of KRAS^G12C^-inhibitor treatment.

We next tested whether ectopic expression of AURKA blocks the induction of Hh signal-related genes and the re-expression of KRAS in cancer cells treated with a KRAS^G12C^ inhibitor. Ectopic expression of AURKA (Fig. 4L) countered the effect of ARS-1620 on induction of GLI-1 (Fig. 4M) and KRAS (Fig. 4N) in H358 cells. Ectopic expression of AURKA also blocked ARS-1620–induced KRAS promoter activity in cancer cells (Fig. 4O). Collectively, our data suggest that downregulation of AURKA in KRAS^G12C^ inhibitor–treated cancer cells induces Hh signaling and re-expression of KRAS.

### Inhibition of Hh signals blocks re-expression of KRAS and reactivation of ERK in KRAS^G12C^ inhibitor–treated cancer cells

**Fig. 5:**
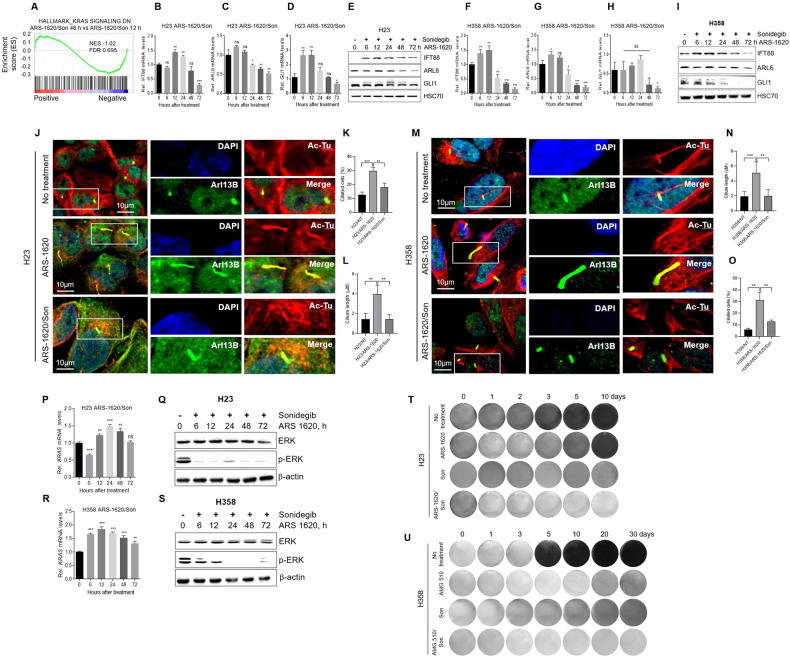
Inhibition of hedgehog signal suppresses re-expression of KRAS and reactivation of ERK in KRAS^G12C^ inhibitor–treated lung cancer cells. H358 cells were co-treated with 10 μM ARS-1620 and 10 μM Smo inhibitor sonidegib for the indicated times. **A** Transcriptome profiles were analyzed by RNA-seq as described in Fig. 1 legends. Gene set enrichment plot of KRAS signaling negatively enriched in cells co-treated with ARS-1620 and the Hedgehog signal inhibitor sonidegib for 48 h versus cells treated with ARS-1620 for 48 h. **B**–**I** The Smo inhibitor sonidegib suppressed ARS-1620-induced Hedgehog signaling in both H23 (**B**–**E**) and H358 cells (**F**–**I**). Time-dependent changes in IFT88 (**B**, **F**), ARL6 (**C**, **G**), and GLI-1 mRNA levels (**D**, **H**) were determined by qRT-PCR amplification. Fold change in expression level was calculated relative to the values at time 0 for each cell. The graphs are mean ± standard deviation of three independent experiments (one-way ANOVA, **P < 0.01; ***P < 0.001). Time-dependent changes in IFT88, ARL6 and GLI-1 shown in H23 (**E**) and H358 cells (**I**) were determined by western blot analysis. **J**–**O** The Smo inhibitor sonidegib suppresses ARS-1620-induced primary cilia formation in both H23 (**J**–**L**) and H358 cells (**M**–**O**). Representative confocal microscopy images of H23 (**J**) and H358 cells (**M**) stained for acetylated tubulin (Ac-Tu, red), Arl13B (green), and DAPI (blue). **K**, **L**, **N**, **O** Graphs depict the percentages of ciliated H23 (**K**) and H358 cells (**N**) and average length of cilia of H23 (**L**) and H358 cells (**O**) and are presented as the mean ± standard deviation (*n* = 150 pooled from three independent experiments). Student’s t-tests, *P < 0.05; **P < 0.01; ***P < 0.001. **P**–**S** The Smo inhibitor sonidegib suppresses ARS-1620–induced re-expression and reactivation of ERK signal in lung cancer cells. Time-dependent changes in *KRAS* mRNA levels in H23 (**P**) and H358 cells (**R**) were determined by qRT-PCR amplification. Fold change in expression level was relative to the values at time 0 for each cell. The graphs are mean ± standard deviation of three independent experiments (one-way ANOVA, **P < 0.01; ***P < 0.001). Time-dependent changes in ERK phosphorylation in H23 (**Q**) and H358 cells (**S**) were determined by western blot analysis (**T**, **U**). The Smo inhibitor sonidegib suppressed the generation of cells resistant to KRAS^G12C^ inhibitors. At the indicated times, H23 (**T**) and H358 cells (**U**) were stained with crystal violet and photographed. Three independent experiments were performed and representative images are shown.

Considering the role of Hh signaling in the expression of genes involved in cell proliferation [30, 31], it is possible that upregulation of Hh signaling may play a role in re-expression of KRAS in KRAS^G12C^ inhibitor–treated cancer cells. To confirm this hypothesis, RNA-seq was conducted using RNA from H358 cells co-treated with ARS-1620 and Hh signal inhibitor sonidegib. Hallmark GSEA showed that, while KRAS signaling was positively enriched in H358 cells treated with ARS-1620 for 48 h compared with cells treated with ARS-1620 for 12 h (Fig. 2F, G), the KRAS signaling was negatively enriched in cells co-treated with ARS-1620 and sonidegib for 48 h compared with cells co-treated for 12 h (Fig. 5A, Table S4 and Table S5). We confirmed the RNA-seq results by conducting qRT-PCR amplification and western blot assays. Sonidegib inhibited the induction of genes involved in Hh signaling in ARS-1620-treated H23 (Fig. 5B–E and Supplemental Material 5) and H358 cells (Fig. 5F–I and Supplemental Material 5). This drug also suppressed the formation of primary cilia in H23 (Fig. 5J–L) and H358 cells (Fig. 5M–O) after ARS-1620 treatment. Importantly, sonidegib blocked the re-expression of KRAS and reactivation of ERK in both H23 (Fig. 5P, Q and Supplemental Material 5) and H358 cells (Fig. 5R, S and Supplemental Material 5), which were detected after ARS-1620 treatment (Fig. 1). In a crystal violet proliferation assay, when cells were treated with ARS-1620 (Fig. 5T) or AMG 510 (Fig. 5U) only, cells had an initial inhibition followed by proliferation (Fig. 5T, U). However, combined treatment of a KRAS^G12C^ inhibitor with sonidegib inhibited cell growth until the end of the experiment (Fig. 5T, U). Even though sonidegib alone inhibited the formation of primary cilia and the expression of GLI target genes and thus decreased phosphorylation of ERK and cell growth in both H23 and H358 cells (Fig. S4 and Supplemental Material 6), combination of sonidegib with KRAS^G12C^ inhibitor showed much better inhibitory effect on ERK phosphorylation and cell proliferation than sonidegib alone (Fig. 5, Fig. S4, Supplemental Material 5 and 6). Taken together, our data suggest that an Hh signal is required for the re-expression of KRAS, reactivation of ERK, and sonidegib blocks the generation of acquired resistance in cancer cells after treatment with a KRAS^G12C^ inhibitor.

### GLI-1 is responsible for reactivation of KRAS induced by KRAS^G12C^ inhibitor in cancer cells

**Fig. 6:**
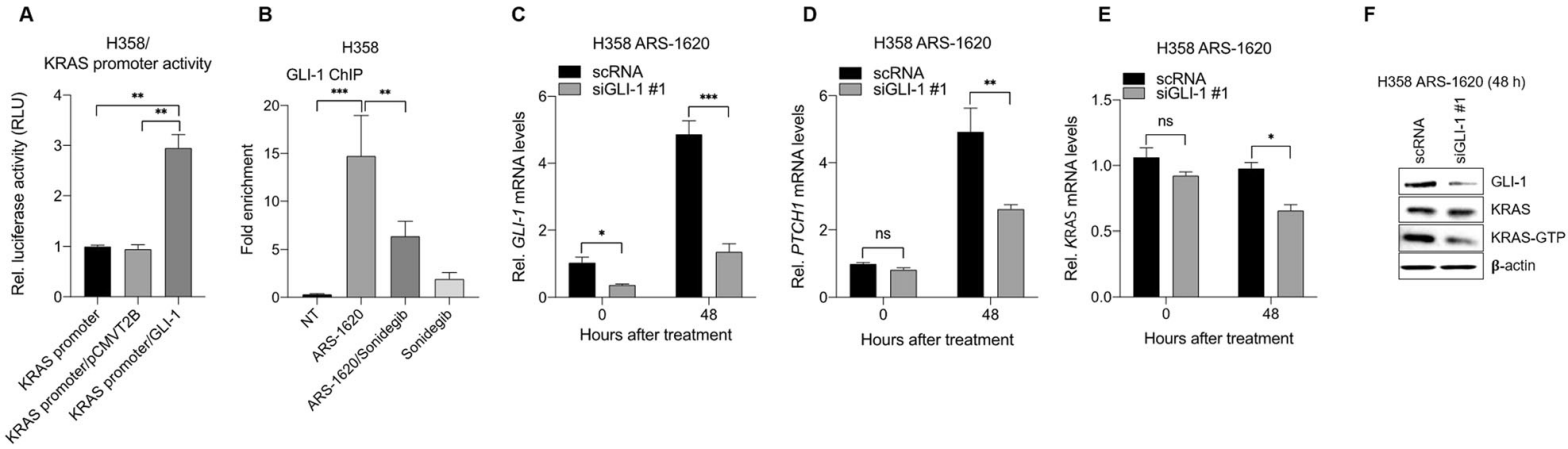
GLI-1 is required to induce KRAS expression in KRAS^G12C^ inhibitor–treated cells. **A** Ectopic expression of GLI-1 activates KRAS promoters. H358 cells were co-transfected with the luciferase reporter vector pEZX-PG04.1/KRAS promoter and pCMV6-Entry GLI-1 expression vector, with an empty vector used as a control. The fold change in luciferase activity was calculated relative to that of cells transfected with the pEZX-PG04.1/KRAS reporter vector only. The graphs are mean ± standard deviation of three independent experiments (one-way ANOVA, ***P < 0.001). **B** GLI-1 binds to the KRAS promoter region. H358 cells were co-treated with 10 μM ARS-1620 and 10 μM sonidegib for 24 h. Chromatin immunoprecipitation–qPCR amplification was conducted to quantitate binding of GLI-1 to KRAS promoter. Fold-enrichment of the KRAS promoter was calculated relative to isotype control. The values represent mean ± standard deviation (*n* = 3) (one-way ANOVA, ***P < 0.001). **C**–**F** Inhibition of GLI-1 attenuates ARS-1620–induced accumulation of KRAS-GTP. H358 cells were transfected with siRNA #1 against GLI-1, followed by 10 μM ARS-1620 treatment for 48 h. Scrambled scRNA was used as a control. Changes in the GLI-1 (**C**, **F**), PTCH-1 (**D**), and KRAS levels (**E**, **F**) were determined by qRT-PCR and Western blot analysis. Fold change in expression levels was calculated relative to the values of scRNA-treated cells at time 0. The graphs are mean ± standard deviation of three independent experiments (one-way ANOVA, *P < 0.01; ***P < 0.001).

Because transcriptional programs induced by Hh signal depend on the GLI transcription factor [32, 33], we tested whether GLI-1 mediates the re-expression of KRAS in cancer cells treated with a KRAS^G12C^ inhibitor. GLI-1 binds to the GACCACCCA motif to regulate transcription of target genes [31]. A search for transcription-factor binding sites using online software (MatInspector) revealed no putative binding sites for GLI-1 within the *KRAS* promoter region. However, ectopic expression of GLI-1 increased *KRAS* promoter activity in cancer cells without KRAS^G12C^-inhibitor treatment (Fig. 6A), suggesting that GLI-1 plays a role in enhancing KRAS-promotor activity. To determine whether Gli binds to the *KRAS* promoter in vivo, we performed chromatin immunoprecipitation (ChIP) assays in H358 cells transfected with a GLI-1–expressing vector without KRAS^G12C^-inhibitor treatment. Chromatin was sonicated into fragments and precipitated using isotype control or anti-GLI-1 antibody. The precipitated DNA was subjected to PCR using primers designed to amplify four regions covering the *KRAS* promoter (Fig. S5A and S5B). GLI-1 bound only to the first region of the *KRAS* promoter (−1,262 to −1,059, amplified by KRAS-P1) (Fig. S5A, S5B and Supplemental Material 7). We next conducted ChIP assay in H358 cells treated with ARS-1620 using PCR primers (KRAS-P5) amplifying 117-bp fragment (-1,205 to -1,089) within the first region of *KRAS* promoter. ARS-1620 treatment increased the binding of GLI-1 to the first region of *KRAS* promoter, which was inhibited by sonidegib treatment (Fig. 6B). This indicates that ARS-1620 enhances the binding of GLI-1 to the *KRAS* promoter. To determine whether GLI-1 is required for ARS-1620–induced KRAS re-expression, H358 cells transfected with siRNA against *GLI-1* were treated with ARS-1620, and KRAS expression was examined. Inhibition of *GLI-1* (Fig. 6C and Fig. S6A) attenuated ARS-1620–induced expression of *PTCH1* (Fig. 6D and Fig. S6B) and *KRAS* mRNA (Fig. 6E and Fig. S6C) and accumulation of KRAS-GTP (Fig. 6F and Supplemental Material 8) in H358 cells. Collectively, these data indicate that ARS-1620 enhances GLI-1 binding to the KRAS promoter in cancer cells.

## Discussion

KRAS^G12C^ inhibitors have shown promising activity in cancers harboring KRAS^G12C^ [13, 14], but the acquisition of resistance to KRAS^G12C^ inhibitors limits the clinical efficacy of these inhibitors [15]. Previously, it has been reported that re-expression of KRAS and reactivation of MAPK pathway can induce acquired resistance against KRAS^G12C^ inhibitors [18, 19]. However, the underlying mechanisms of re-expression of KRAS and reactivation of MAPK pathway have not yet been clarified. In this report, we demonstrate that Hh signal contributes to the re-expression of KRAS and reactivation of ERK and, thus, induction of acquired resistance against KRAS^G12C^ inhibitor in cancer cells. We revealed that KRAS^G12C^ inhibitors enhance the formation of primary cilia and Hh signaling, and that inhibition of Hh signaling blocks re-expression of KRAS and reactivation of ERK in lung cancer cells treated with a KRAS^G12C^ inhibitor. In addition, we provide a molecular basis for how Hh signals induce re-expression of KRAS in cells treated with a KRAS^G12C^ inhibitor, implying that combined treatment of KRAS^G12C^ inhibitors and Hh signal inhibitors may overcome acquired resistance in cancer patients harboring KRAS^G12C^.

The Hh signal activates transcription factor GLI, which in turn induces expression of Hh target genes [26]. Among the target genes is *GLI-1* itself [31, 36], which represents a reliable marker for Hh pathway activation [37, 38]. We found that KRAS^G12C^-inhibitor treatment enhanced expression of transcription factor GLI-1 and other GLI-1-target genes, indicating activation of the Hh signal. The transcription factor GLI binds to the consensus sequence GACCACCCA motif to activate Hh target genes [31]. However, GLI can bind to non-canonical GLI-binding sites with relatively low affinity, still leading to strong transcriptional activation [39–41]. Although we could not detect the canonical GLI consensus sequence within the promoter region of the KRAS, GLI bound to the promoter region of KRAS and enhanced KRAS promoter activity in KRAS^G12C^ inhibitor–treated cells. These results suggest that GLI1 induces the expression of KRAS by binding to a functional non-canonical GLI-binding site (between -1262 and -1059) within the KRAS promoter region. In addition, inhibition of GLI-1 using siRNA suppressed re-expression of KRAS in cells treated with a KRAS^G12C^ inhibitor, indicating that GLI-1 is required for re-expression of KRAS in such cells.

Activation of GLI is triggered by the binding of Hh ligands to their receptor PTCHs [42]. The binding of Hh ligands to PTCHs activates Smo [43, 44] and subsequently GLI to upregulate Hh target genes, including factors involved in cell proliferation, survival, self-renewal, and invasiveness [30, 31, 45]. RNA-seq analysis revealed that treatment with a KRAS^G12C^ inhibitor did not increase the expression levels of Hh ligands, suggesting that GLI-1 may be activated in the absence of Hh ligands. Several recent studies also have shown that GLI can be activated by oncogenic pathways such as KRAS and TGF-β, independently of the Hh ligand-PTCH1-Smo route in cancer cells [46–48]. However, the Smo inhibitor sonidegib suppressed induction of GLI-1 and re-expression of KRAS in KRAS^G12C^ inhibitor–treated cells, suggesting that activation of GLI-1 and re-expression of KRAS occur in a Smo-dependent manner. Further studies are required to clarify the underlying mechanism for activation of GLI-1 in KRAS^G12C^ inhibitor–treated cells.

Transduction of the Hh signal requires the formation of primary cilia, microtubule-based sensory structures [42, 49], which occurs at the G0 or G1 phase of the cell cycle [34, 50]. Cell cycle–related kinase AURKA at the basal body of primary cilia stimulates HDAC6-mediated deacetylation and destabilization of microtubules and plays an essential role in the disassembly and resorption of primary cilia [34, 51]. AURKA is activated in the late G2 phase and, after mitosis, most AURKA proteins undergo degradation during the G1 phase [35]. Considering the role of primary cilia in the transduction of Hh signals [42, 49] and cell cycle arrest of cells treated with a KRAS^G12C^ inhibitor at G0/G1 phase [18], it is possible that downregulation of AURKA in KRAS^G12C^ inhibitor–treated cells may be responsible for the induction of Hh signaling and re-expression of KRAS. Here we provide evidence supporting a key role for AURKA in the induction of Hh signaling and re-expression of KRAS in cells treated with a KRAS^G12C^ inhibitor. First, expression levels of AURKA decreased after treatment with a KRAS^G12C^ inhibitor. Second, inhibition of AURKA using siRNA increased the expression of GLI-1 target genes in the absence of treatment with a KRAS^G12C^ inhibitor. Third, inhibition of AURKA using siRNA also increased the expression of KRAS in the absence of KRAS^G12C^-inhibitor treatment. Fourth, ectopic expression of AURKA blocked the induction of GLI-1 target genes and KRAS re-expression in cells treated with a KRAS^G12C^ inhibitor. However, our results contradicted those of a previous report suggesting that AURKA is upregulated in cells with acquired resistance to KRAS^G12C^ inhibitors, and that inhibition of AURKA prevents the reactivation of KRAS [18]. Xue et al. [18] suggested that AURKA signals can maintain re-expressed KRAS^G12C^ proteins in an active GTP-bound form, facilitating effector activation and cell cycle progression. Like Xue et al. [18], we found that AURKA levels decreased at 24 h but rebounded at 72 h after treatment with a KRAS^G12C^ inhibitor. Taken together, it is possible that, early point, KRAS^G12C^ inhibitors arrest the cell cycle at G0/G1, thereby decreasing AURKA expression, which induces primary cilia formation, Hh signaling, and KRAS re-expression. However, at a later time point, re-expressed KRAS promotes cell cycle progress and increases AURKA levels, which maintain the re-expressed KRAS^G12C^ in an active GTP-bound form. An improved understanding of the mechanisms involved in AURKA expression in cells treated with a KRAS^G12C^ inhibitor is required to resolve this discrepancy.

In summary, our study shows that KRAS^G12C^ inhibitors induce the formation of primary cilia and activate Hh signaling, which is responsible for re-expression of KRAS and reacquired resistance against KRAS^G12C^ inhibitors in cancer cells. Several Hh signal inhibitors are used in clinical trials for multiple types of cancers. Most of the efforts to inhibit Hh signaling have been directed at the development of Smo inhibitors. The US Food and Drug Administration and European Medicines Agency have approved two Smo inhibitors, vismodegib and sonidegib, for the treatment of locally advanced or metastatic basal cell carcinoma [52, 53]. We provide evidence that sonidegib suppresses re-expression of KRAS in cancer cells treated with a KRAS inhibitor. These findings extend our understanding of the mechanisms responsible for acquired resistance in cancer cells treated with KRAS^G12C^ inhibitors and indicate that Smo inhibitors are a therapeutic strategy that can overcome acquired resistance against KRAS^G12C^ inhibitors in cancers.

### Supplementary information

Supplementary Figures

Raw data

Supplementary Table S1

Supplementary Table S2

Supplementary Table S3

Supplementary Table S4

Supplementary Table S5

## Data Availability

The data can be made available upon reasonable request to the corresponding author at the following address: Jeong Woo Park, Department of Biological Sciences, University of Ulsan, Ulsan 44610, Korea. E-mail: jwpark@ulsan.ac.kr.
