# Supplementary material for: Hedgehog signalling is involved in acquired resistance to KRASG12C inhibitors in lung cancer cells
Source: Cell Death Dis. 2024 Jan 16;15(1):56. doi: 10.1038/s41419-024-06436-9 (PMC10789740; doi:10.1038/s41419-024-06436-9)
Supplement: Supplementary file 1 — Supplementary Figures [file 41419_2024_6436_MOESM1_ESM.docx]

**Hedgehog signaling is involved in acquired resistance to KRAS^G12C^ inhibitors in lung cancer cells**

Chaeyoung Lee^1^, Jawoon Yi^2^, Jihwan Park^2^, Byungyong Ahn^3,4^, Young-Wook Won^5,6^, JiHeung Jeon^1^, Byung Ju Lee^1,4^, Wha Ja Cho^1^, Jeong Woo Park^1,4^

^1^Department of Biological Sciences, University of Ulsan, Ulsan 44610, Korea

^2^School of Life Sciences, Gwangju Institute of Science and Technology, Gwangju, 61005, Korea

^3^Department of Food Science and Nutrition, University of Ulsan, Ulsan 44610, Korea

^4^Basic-Clinical Convergence Research Institute, University of Ulsan, Ulsan 44610, Korea

^5^Department of Biomedical Engineering, University of North Texas, Texas 76203-5017, USA

^6^RopheLBio, B102, Seoul Forest M Tower, Seoul 04778, Korea

**Running title**: Hh signaling induces acquired resistance to KRAS^G12C^ inhibitors

Address correspondence to: Wha Ja Cho, wjcho26@ulsan.ac.kr; Jeong Woo Park, jwpark@ulsan.ac.kr


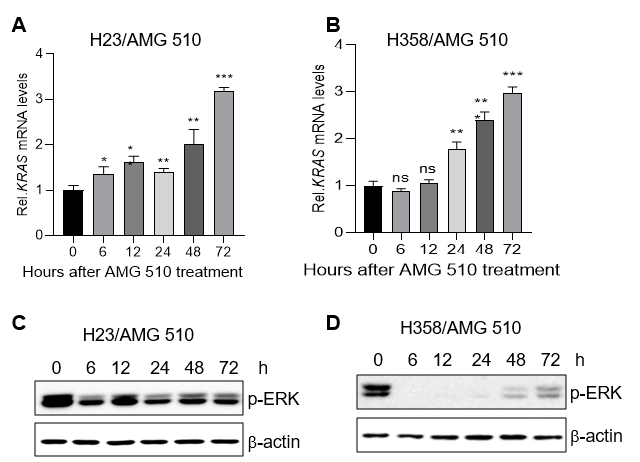


**Supplementary Figure S1**. **The KRAS^G12C^ inhibitor AMG 510 induces re-expression of KRAS and reactivation of ERK signal in lung cancer cells**. (**A**, **C**) H23 and (**B**, **D**) H358 lung cancer cells were treated with 0.1 μM AMG 510 for the indicated times. Time-dependent changes in *KRAS* mRNA levels (**A** and **B**) and ERK phosphorylation levels (**C** and **D**) in H23 (**A** and **C**) and H358 (**B** and **D**) cells were determined by qRT-PCR and western blot analysis, respectively. Fold-change in *KRAS* mRNA expression levels was calculated relative to the values at time 0 for each cell. The graphs are mean ± standard deviation of three independent experiments (one-way ANOVA, ***P* < 0.01; ****P* < 0.001).

**
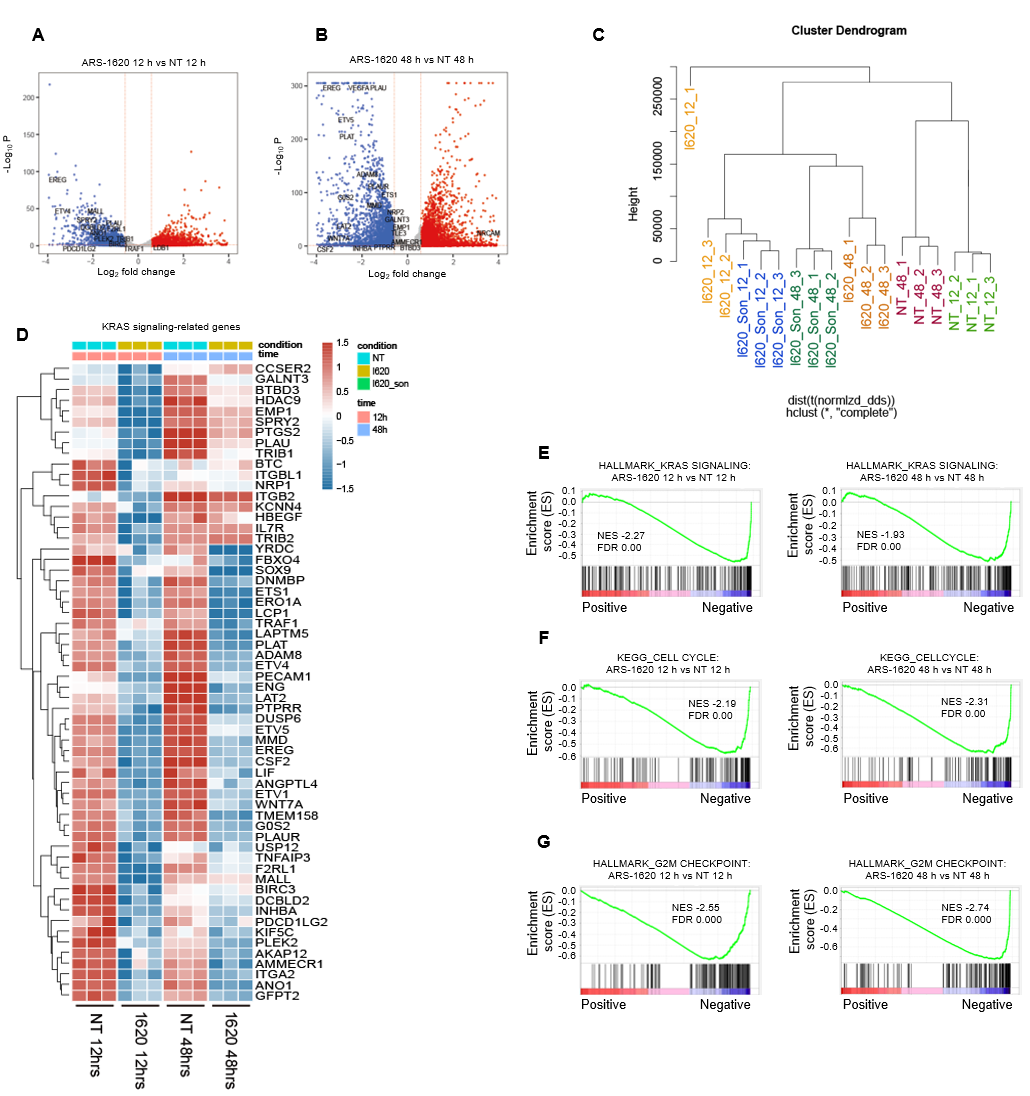
**

**Supplementary Figure S2. RNA-seq analysis of lung cancer cells after treatment with ARS-1620.** H358 cells were treated with a combination of 10 μM ARS-1620 10 μM sonidegib for 12 h or 48 h and their transcriptome profiles were analyzed by RNA-seq. (**A** and **B**) Volcano plots of differentially expressed genes (DEGs) in ARS-1620-treated H358 cells compared with non-treated cells. The y-axis corresponds to the significance level represented with the -log_10_P value, and the x-axis displays the log_2_ (FC) value. Red dots represent significant (adj. P < 0.05 and |Log2 FC| ≥ 1.5) DEGs in (**A**) cells treated with ARS-1620 for 12 h and (**B**) cells treated with ARS-1620 for 48 h. A dotted horizontal line indicates an adj. P = 0.05, and dotted vertical lines indicate a mean |log2FC| of 1.5. (**C**) Unsupervised hierarchical clustering of RNA-seq data from non-treated cells, cells treated with ARS-1620, and cell treated with ARS-1620 plus sonidegib for 12 h or 48 h (n = 3). (**D**) Heat map of KRAS signaling–related genes. (**E**-**G**) Enrichment plots of KRAS signaling (**E**), cell cycle (**F**), and cell checkpoint (**G**) for negatively enriched ARS-1620-treated cells versus non-treated cells at 12 h and 48 h.

**
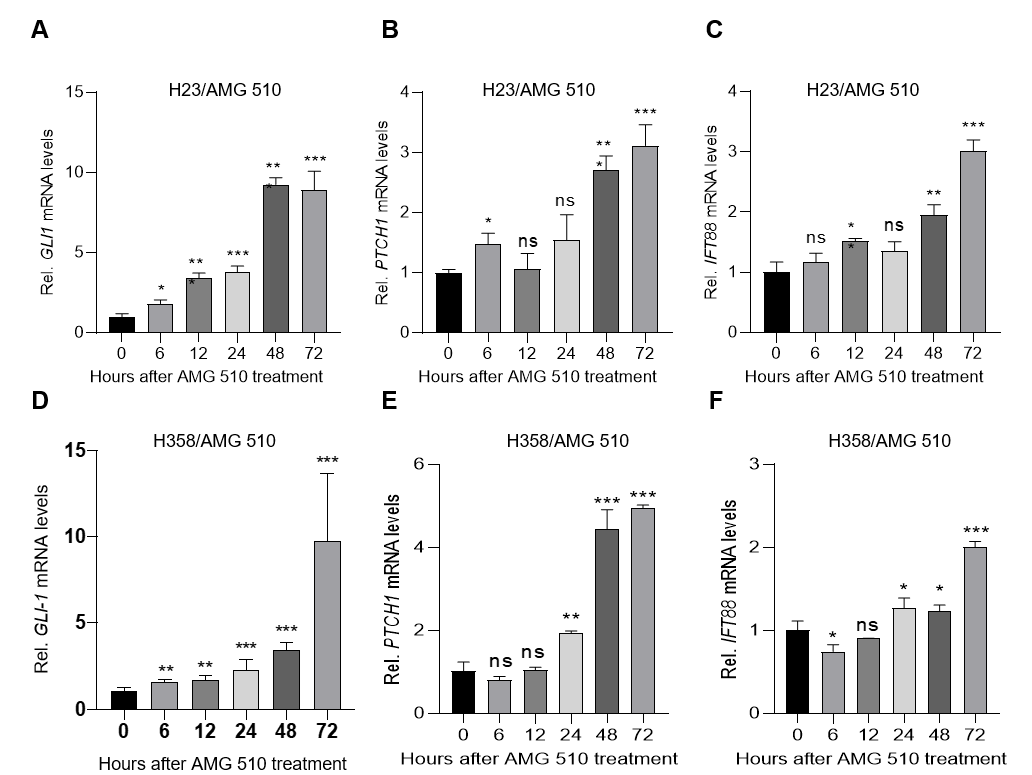
**

**Supplementary Figure S3. The KRAS^G12C^ inhibitor AMG 510 induces Hedgehog signaling in lung cancer cells**. (**A**-**C**) H23 and (**D**-**F**) H358 cells were treated with 0.1 μM AMG 510 for the indicated times. Time-dependent changes in *GLI-1* (**A** and **D**), *PTCH1* (**B** and **E**), and *IFT88* (**C** and **F**) mRNA levels were determined by qRT-PCR. Fold change in expression level was calculated relative to the values at time 0 for each cell. The graphs are mean ± standard deviation of three independent experiments (one-way ANOVA, ***P* < 0.01; ****P* < 0.001).


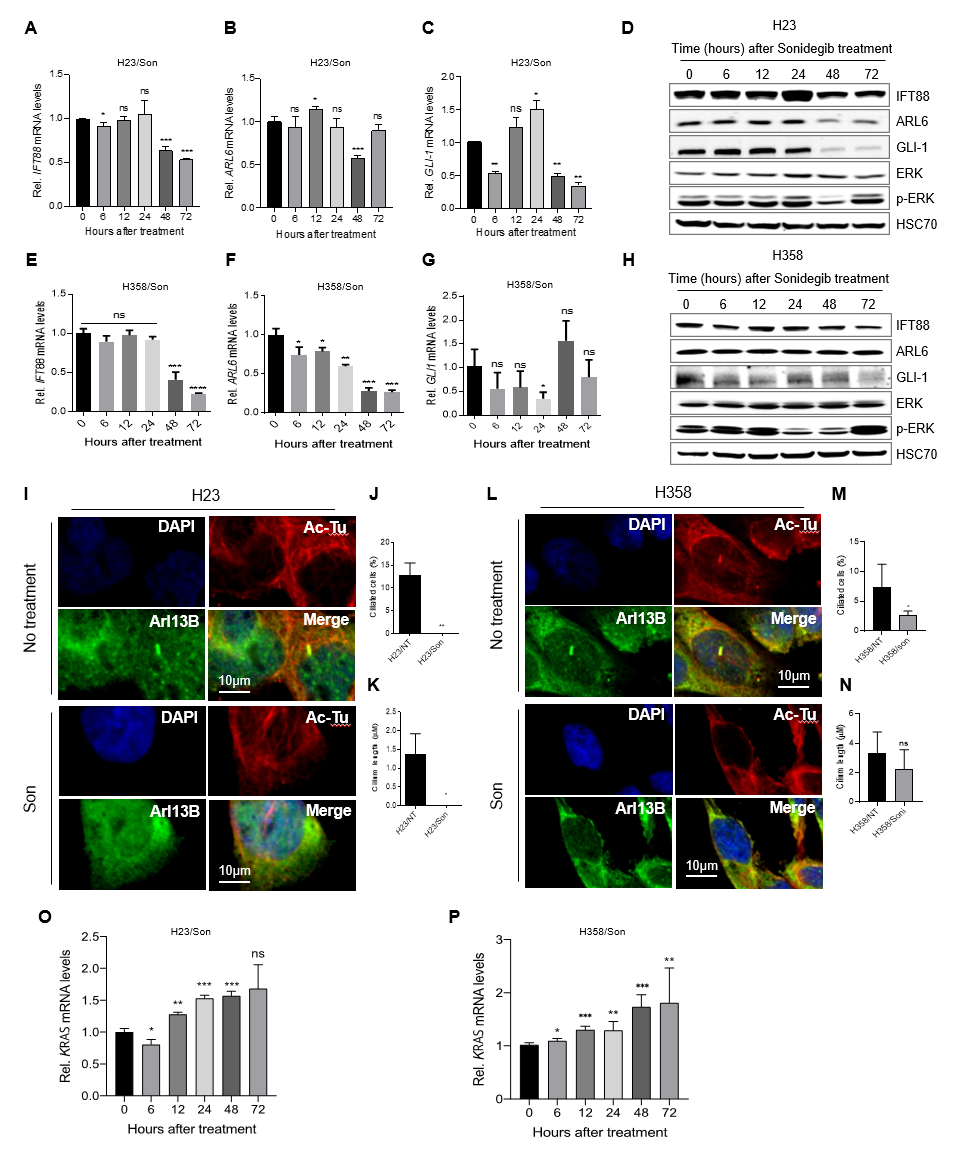


**Supplementary Figure S4**. **Effect of Smo inhibitor sonidegib on the hedgehog signal, expression of KRAS, and activation of ERK in lung cancer cells**. (**A**-**D**) H23 and (**E**-**H**) H358 cells were treated with 10 μM Smo inhibitor sonidegib for the indicated times. Time-dependent changes in (**A, D, E,** and **H**) IFT88, (**B, D, F,** and **H**) ARL6, (**DC, D, G,** and **H**) GLI-1, and (**D** and **H**) ERK levels were determined by qRT-PCR and Western blot analysis. Fold change in expression level was calculated relative to the values at time 0 for each cell. The graphs are mean ± standard deviation of three independent experiments (one-way ANOVA, **P* < 0.05; ***P* < 0.01; ****P* < 0.001; ns, not significant). (**I**–**N**) The Smo inhibitor sonidegib suppresses primary cilia formation in both (**I**–**K**) H23 and (**L**-**N**) H358 cells. Representative confocal microscopy images of (**I**) H23 and (**L**) H358 cells stained for acetylated tubulin (Ac-Tu, red), Arl13B (green), and DAPI (blue). (**J**, **K**, **M**, **N**) Graphs depict the percentages of ciliated (**J**) H23 and (**M**) H358 cells and average length of cilia of (**K**) H23 and (**N**) H358 cells and are presented as the mean ± standard deviation (n = 150 pooled from three independent experiments). Student’s t-tests, **P* < 0.05; ***P* < 0.01; ns, not significant. (**O**, **P**) The effect of Smo inhibitor sonidegib on the expression of *KRAS* mRNA in lung cancer cells. Time-dependent changes in *KRAS* mRNA levels in (**O**) H23 and (**P**) H358 cells were determined by qRT-PCR amplification. Fold change in expression level was relative to the values at time 0 for each cell. The graphs are mean ± standard deviation of three independent experiments (one-way ANOVA, **P* < 0.05; ***P* < 0.01; ****P* < 0.001; ns, not significant).


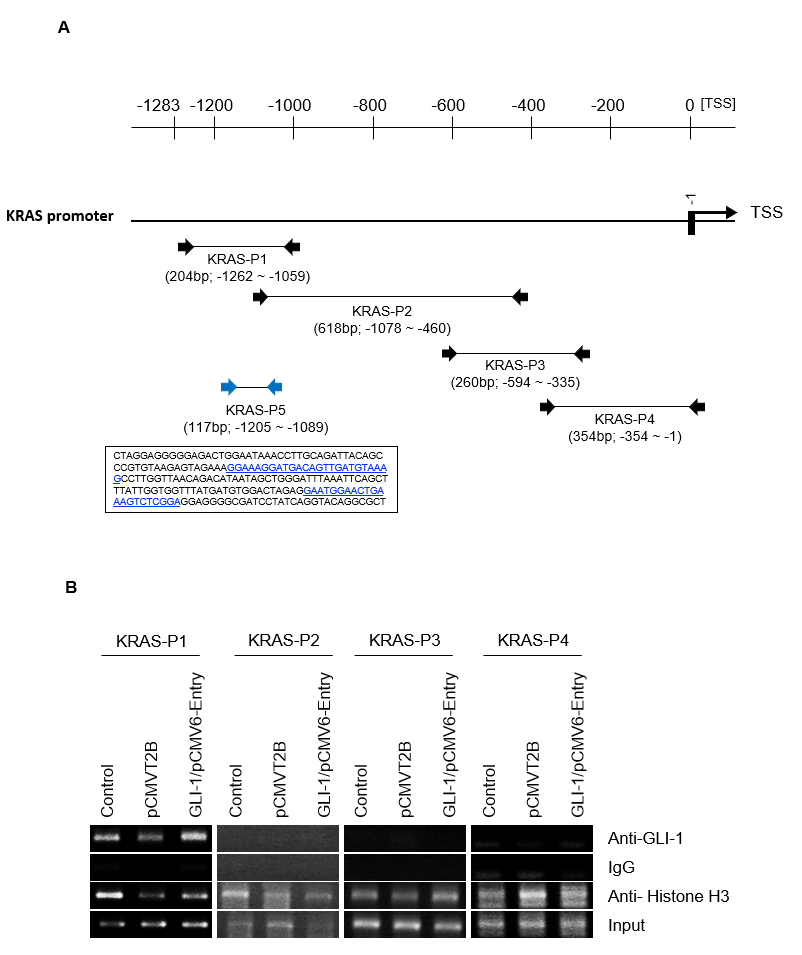


# Supplementary Figure S5. Chromatin immunoprecipitation (ChIP) analysis. (A) The positions of five PCR primer sets on the promoter of the KRAS gene (TSS, transcription start site). The base sequences within the box represent the KRAS promoter region amplified by KRAS-P1. The blue letters within the box represent the positions of KRAS-P5, which was used to generate ChIP data in Fig. 6B. (B) H358 cells were transfected with a GLI-1 expression vector or empty vector. Control cells were not transfected with any vectors. Formaldehyde-cross-linked chromatin from cells was incubated with anti-GLI-1 antibodies or an immunoglobin G isotype control. Total input DNA at a 1:10 dilution and anti-Histone H3 antibodies were used as positive control for the PCR process. Immunoprecipitated DNA was analyzed by PCR amplification with four sets of primers indicated in (A).


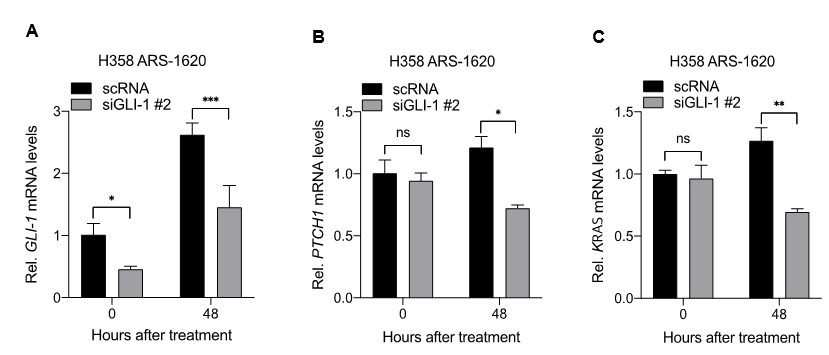


**Supplementary Figure S6. Inhibition of GLI-1 attenuates ARS-1620-induced KRAS expression in lung cancer cells**. H358 cells were transfected with siRNA #2 against GLI-1, followed by 10 μM ARS-1620 treatment for 48 h. Scrambled scRNA was used as a control. Changes in the (**A**) *GLI-1*, (**B**) *PTCH-1*, and (**C**) *KRAS* mRNA levels were determined by qPCR. Fold change in expression levels was calculated relative to the values of scRNA-treated cells at time 0. The graphs are mean ± standard deviation of three independent experiments (one-way ANOVA, **P* < 0.05; ***P* < 0.01; ****P* < 0.001; ns, not significant).
