# Supplementary material for: Hedgehog signalling is involved in acquired resistance to KRASG12C inhibitors in lung cancer cells
Source: Cell Death Dis. 2024 Jan 16;15(1):56. doi: 10.1038/s41419-024-06436-9 (PMC10789740; doi:10.1038/s41419-024-06436-9)
Supplement: Supplementary file 2 — Raw data [file 41419_2024_6436_MOESM2_ESM.pptx]

## Slide 1
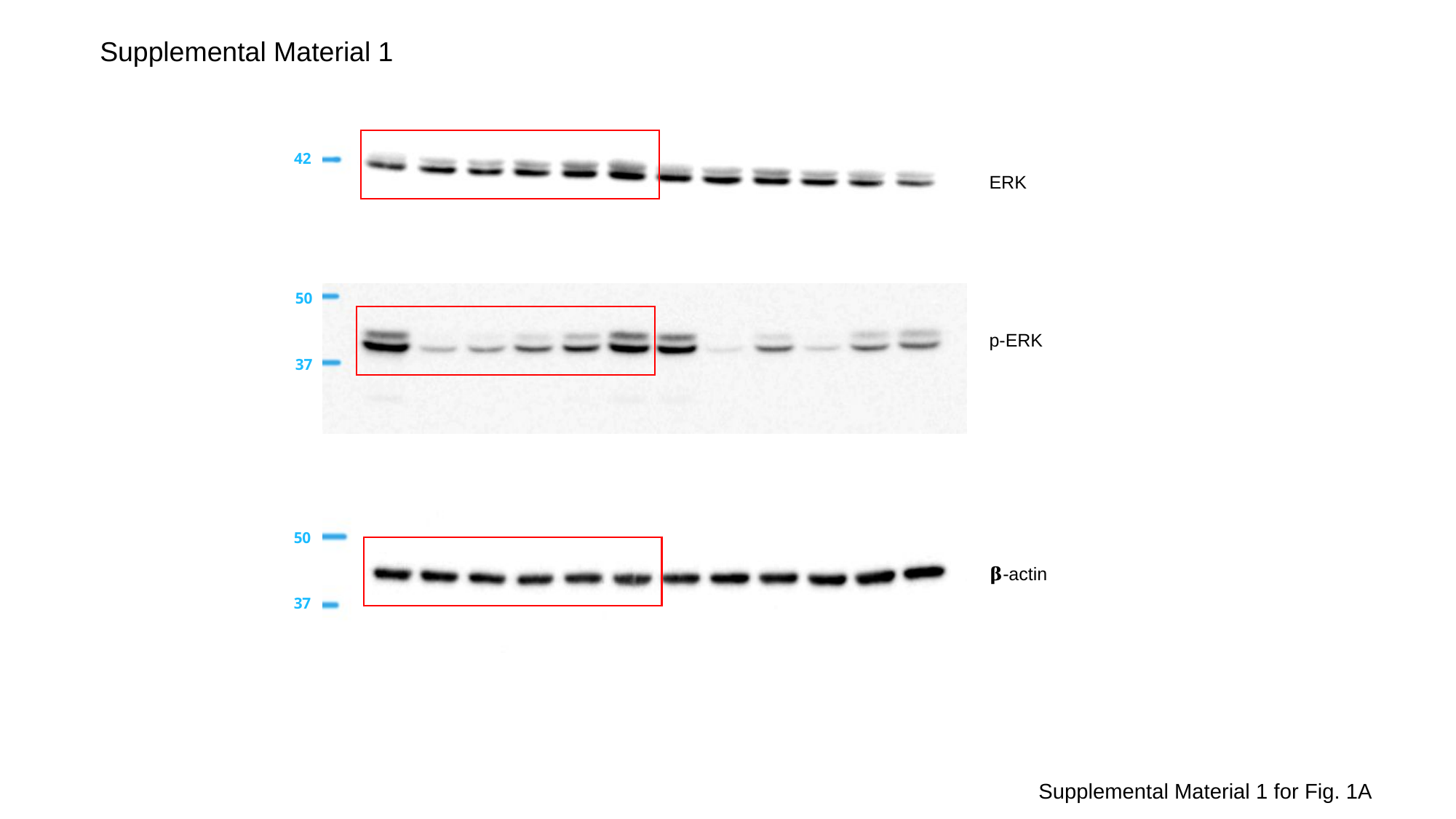

Supplemental Material 1
42
ERK
50
p-ERK
37
50
𝛃-actin
37
Supplemental Material 1 for Fig. 1A

## Slide 2
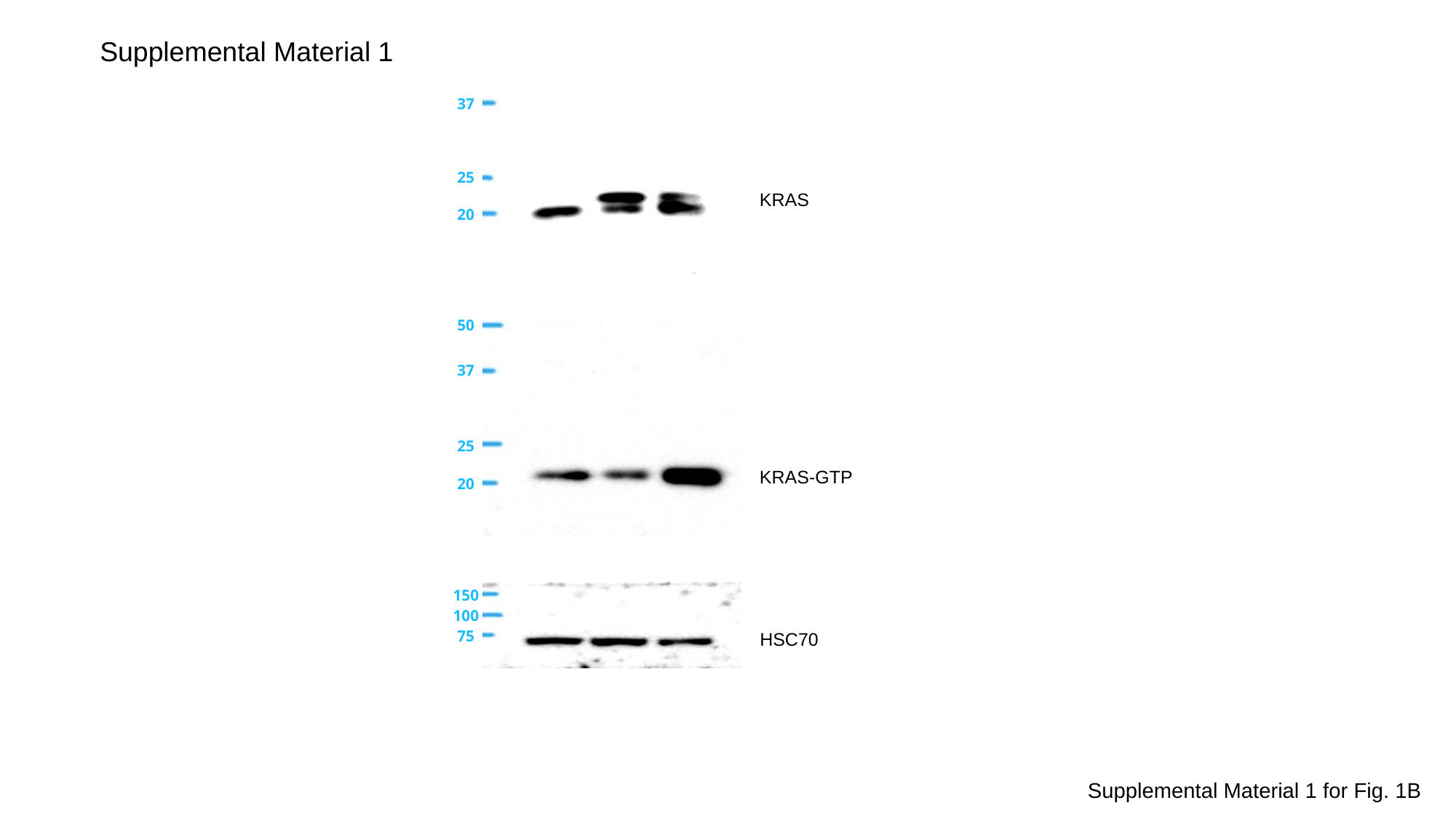

Supplemental Material 1
37
25
KRAS
20
50
37
25
KRAS-GTP
20
150
100
75
HSC70
Supplemental Material 1 for Fig. 1B

## Slide 3
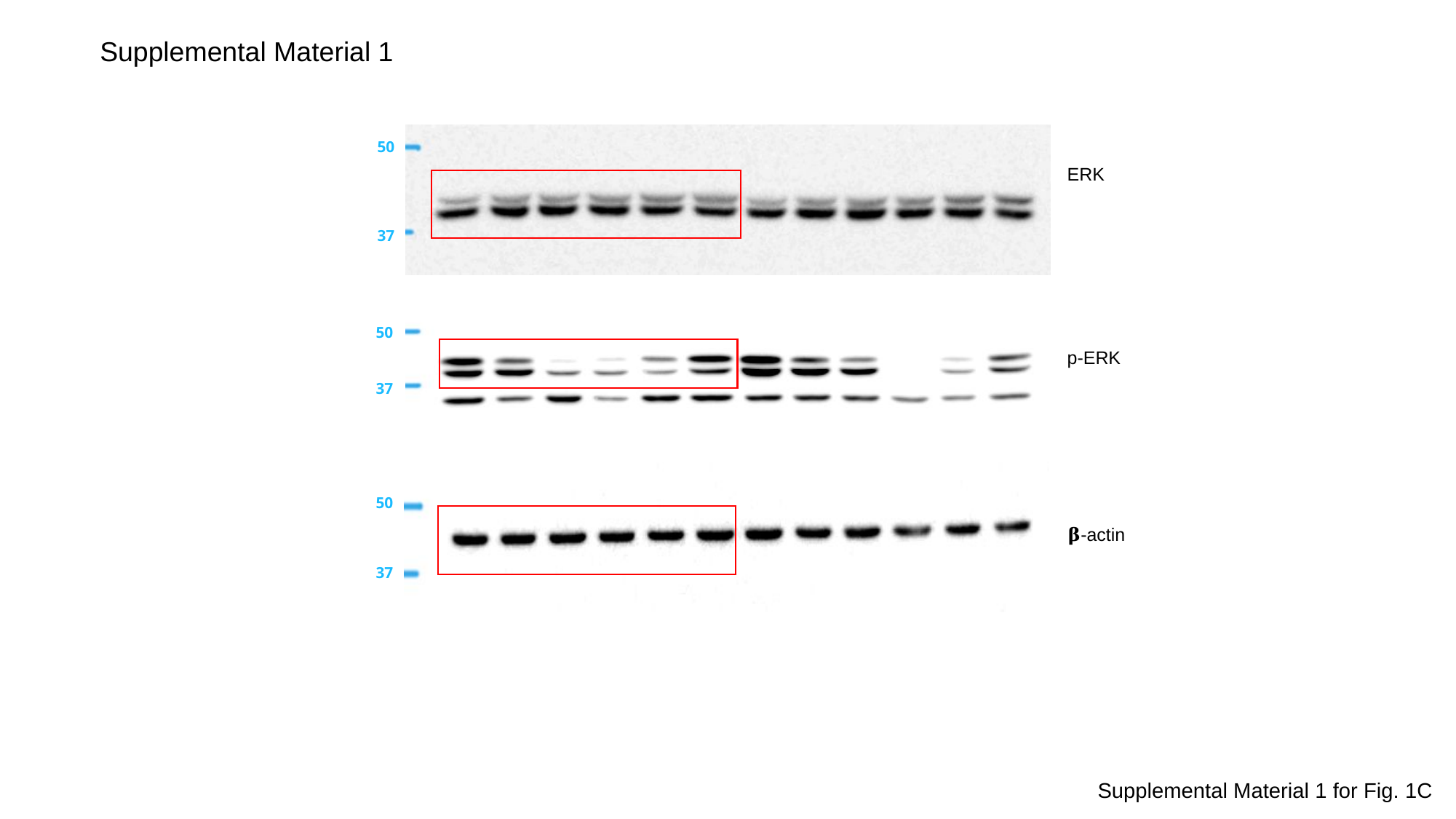

Supplemental Material 1
50
ERK
37
50
p-ERK
37
50
𝛃-actin
37
Supplemental Material 1 for Fig. 1C

## Slide 4
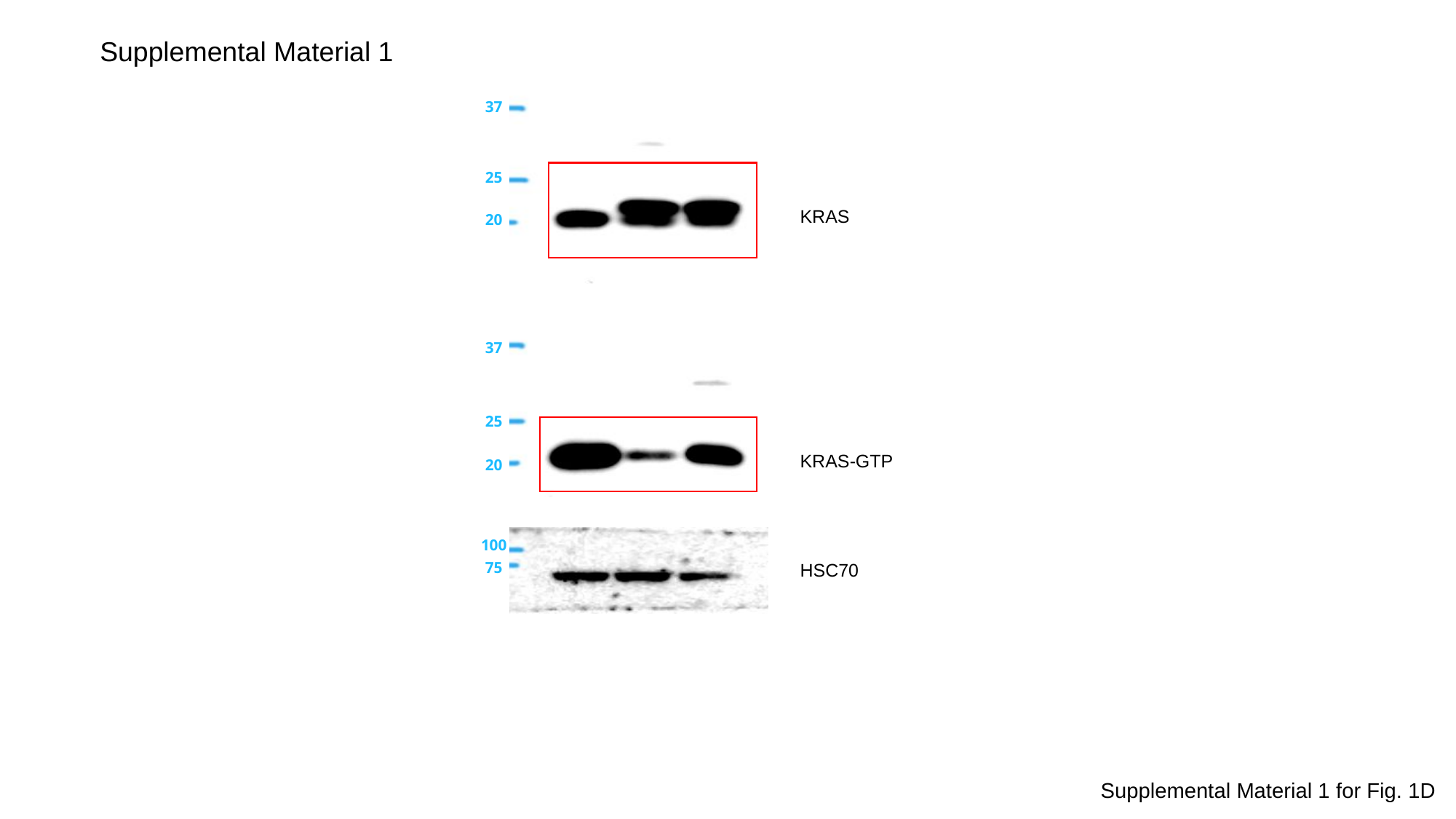

Supplemental Material 1
37
25
KRAS
20
37
25
KRAS-GTP
20
100
75
HSC70
Supplemental Material 1 for Fig. 1D

## Slide 5
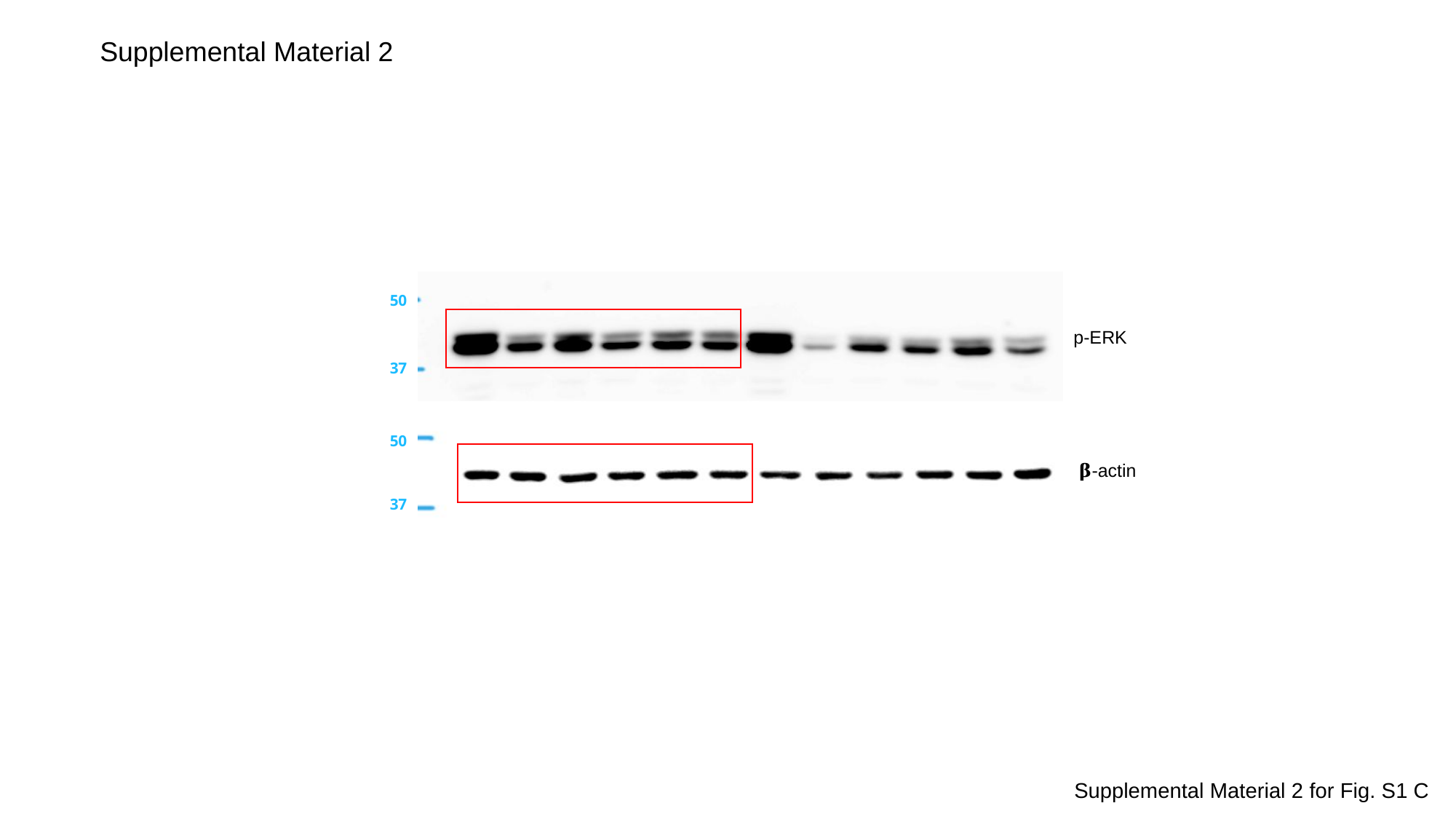

Supplemental Material 2
50
p-ERK
37
50
𝛃-actin
37
Supplemental Material 2 for Fig. S1 C

## Slide 6
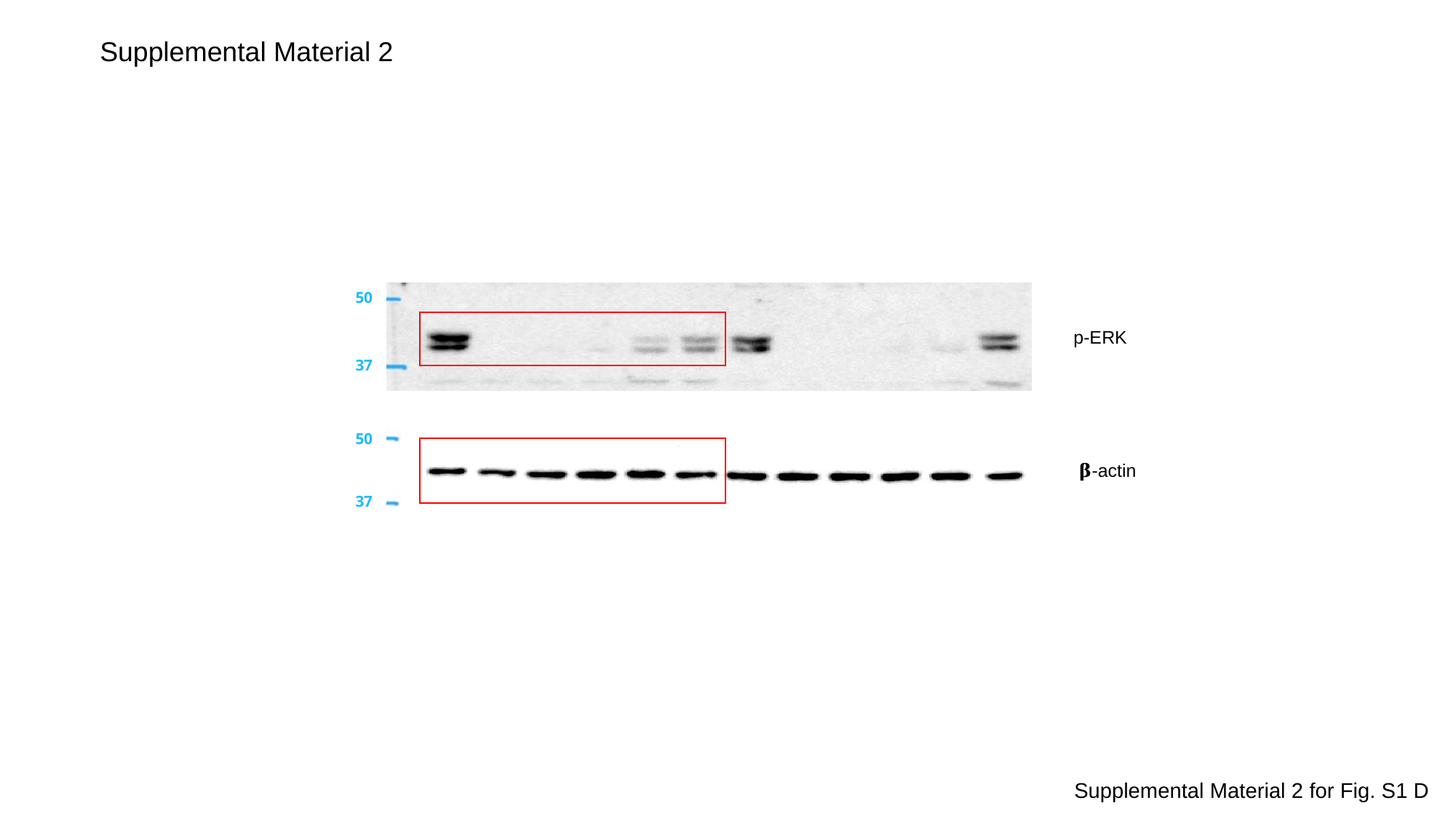

Supplemental Material 2
50
p-ERK
37
50
𝛃-actin
37
Supplemental Material 2 for Fig. S1 D

## Slide 7
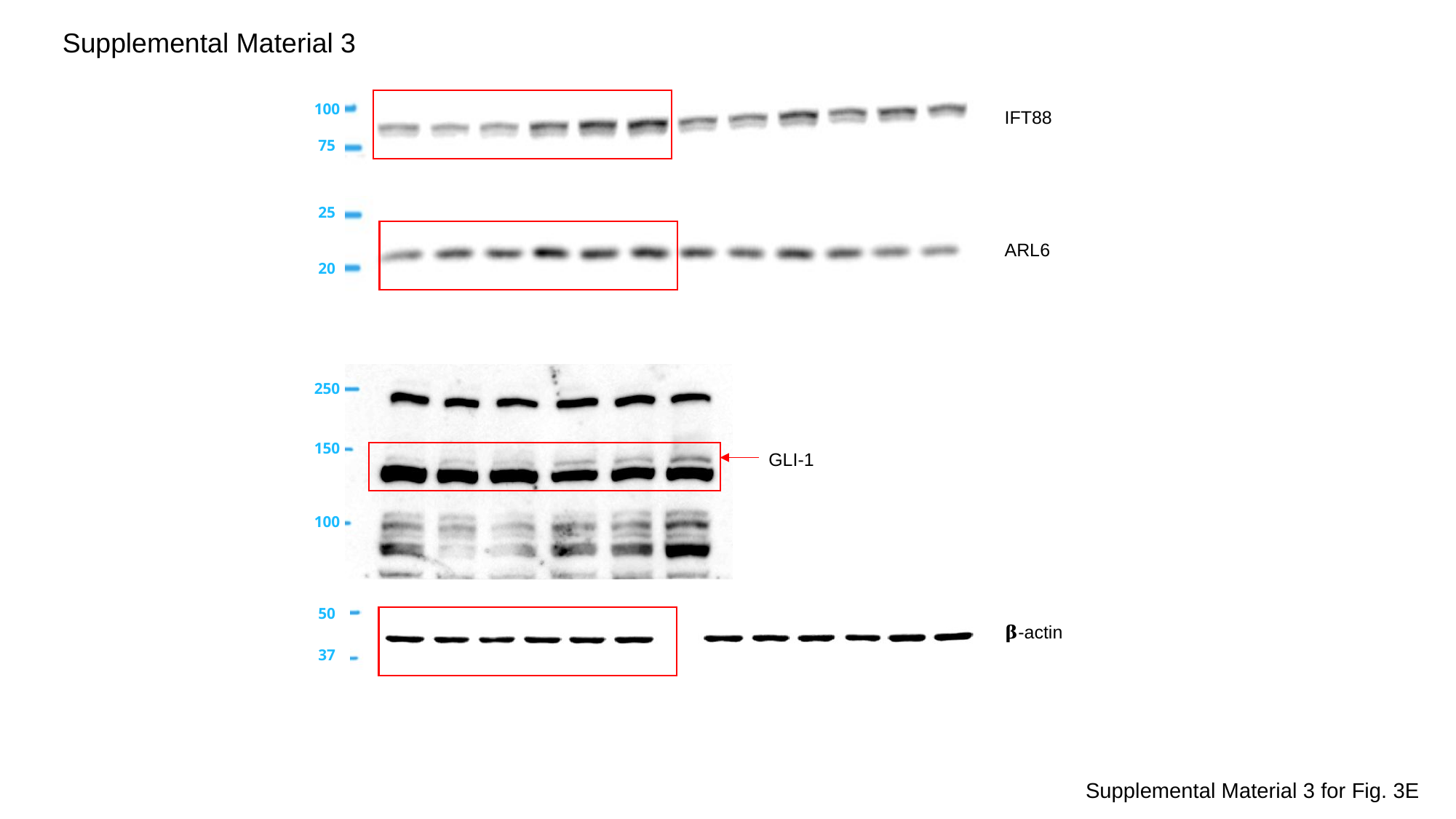

Supplemental Material 3
100
IFT88
75
25
ARL6
20
250
150
GLI-1
100
50
𝛃-actin
37
Supplemental Material 3 for Fig. 3E

## Slide 8
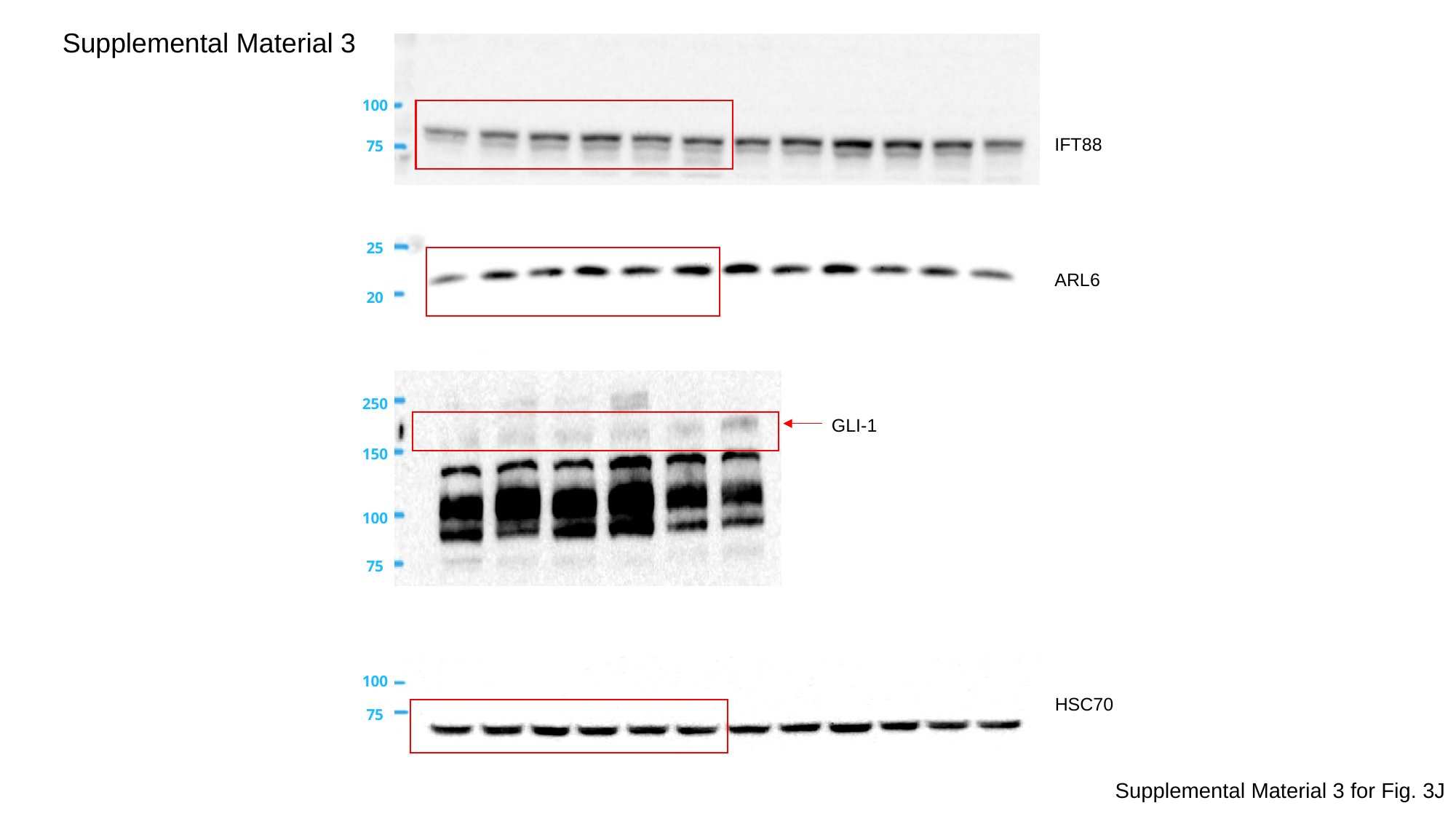

Supplemental Material 3
100
IFT88
75
25
ARL6
20
250
GLI-1
150
100
75
100
HSC70
75
Supplemental Material 3 for Fig. 3J

## Slide 9
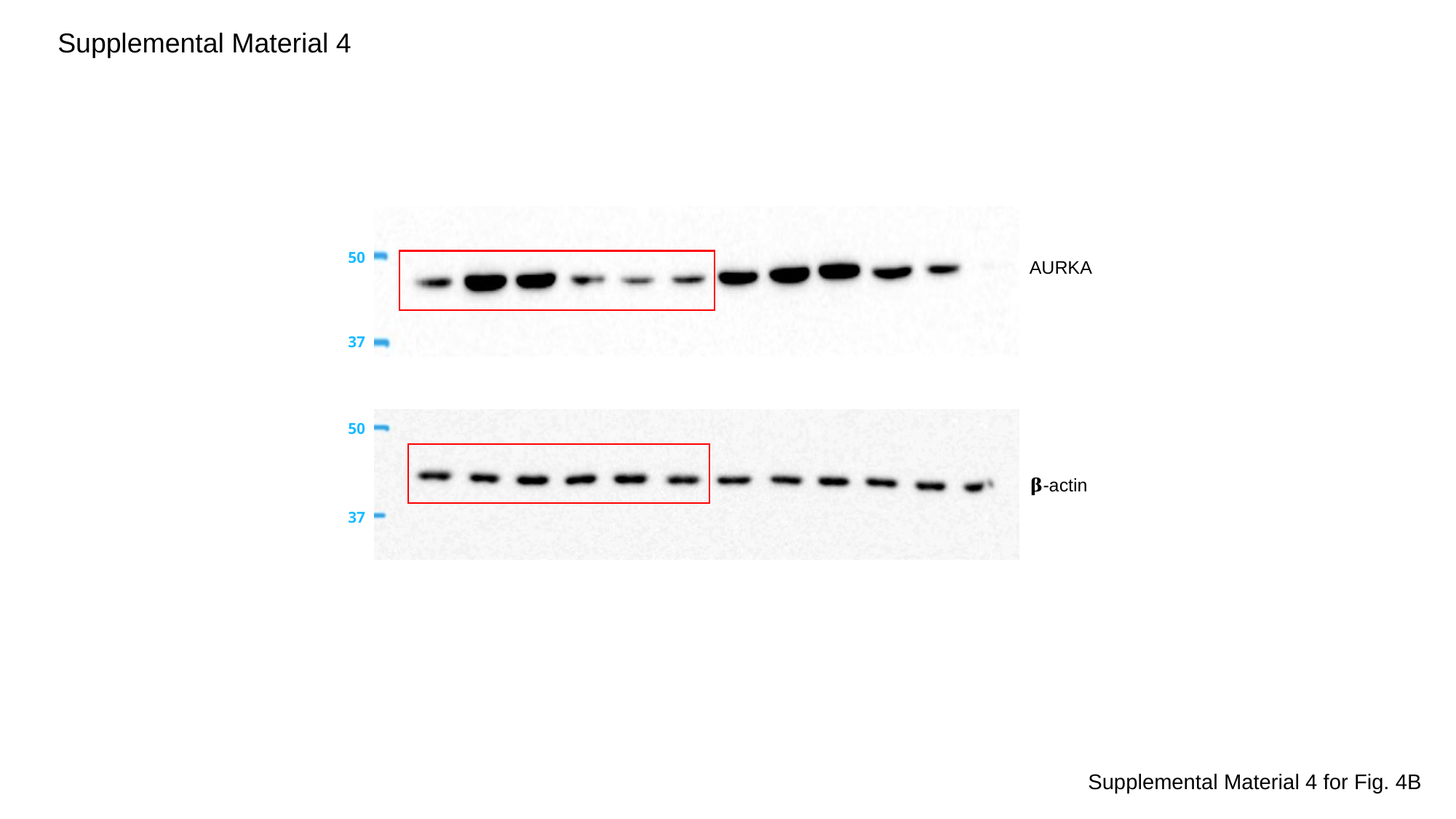

Supplemental Material 4
50
AURKA
37
50
𝛃-actin
37
Supplemental Material 4 for Fig. 4B

## Slide 10
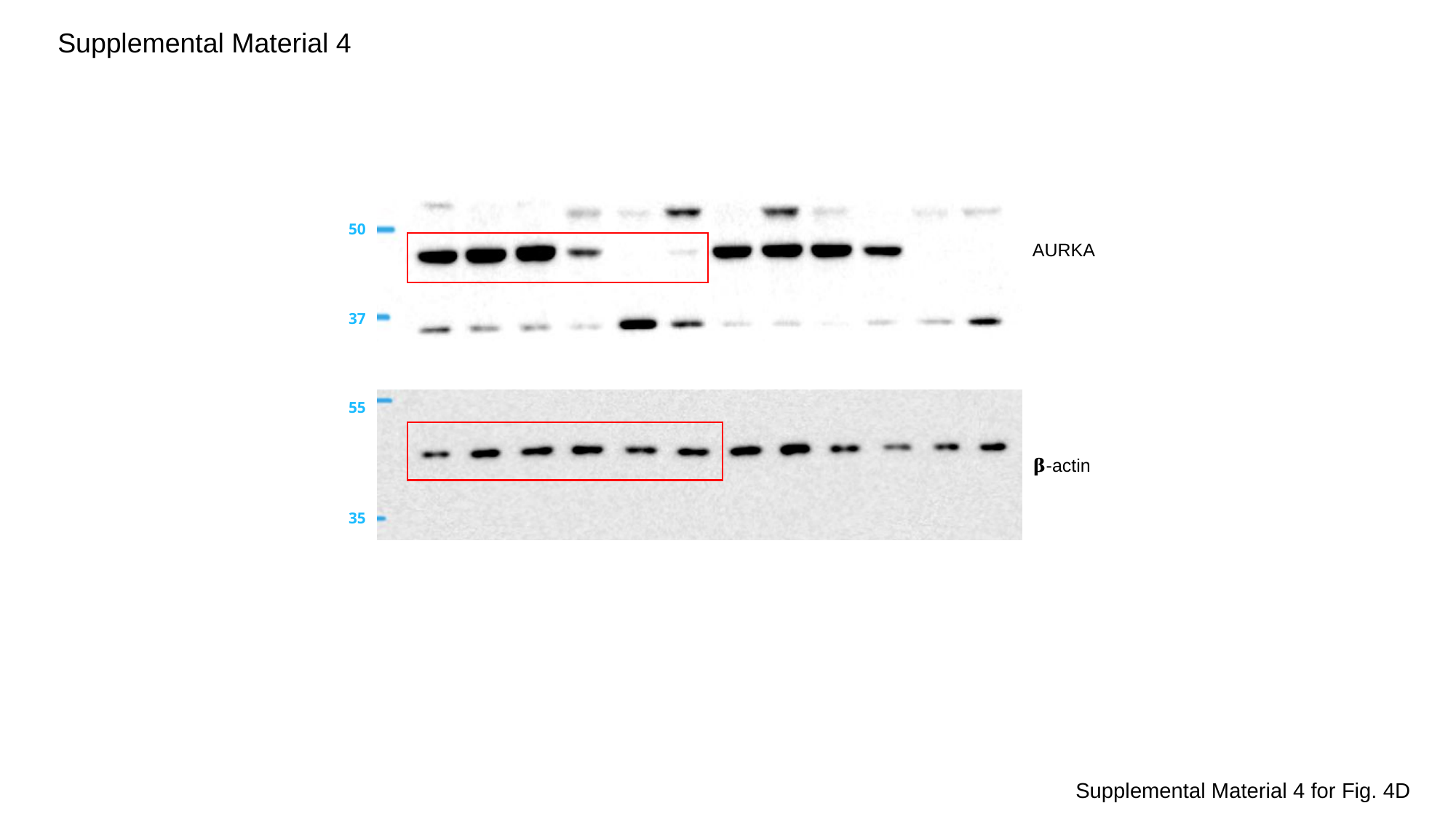

Supplemental Material 4
50
AURKA
37
55
𝛃-actin
35
Supplemental Material 4 for Fig. 4D

## Slide 11
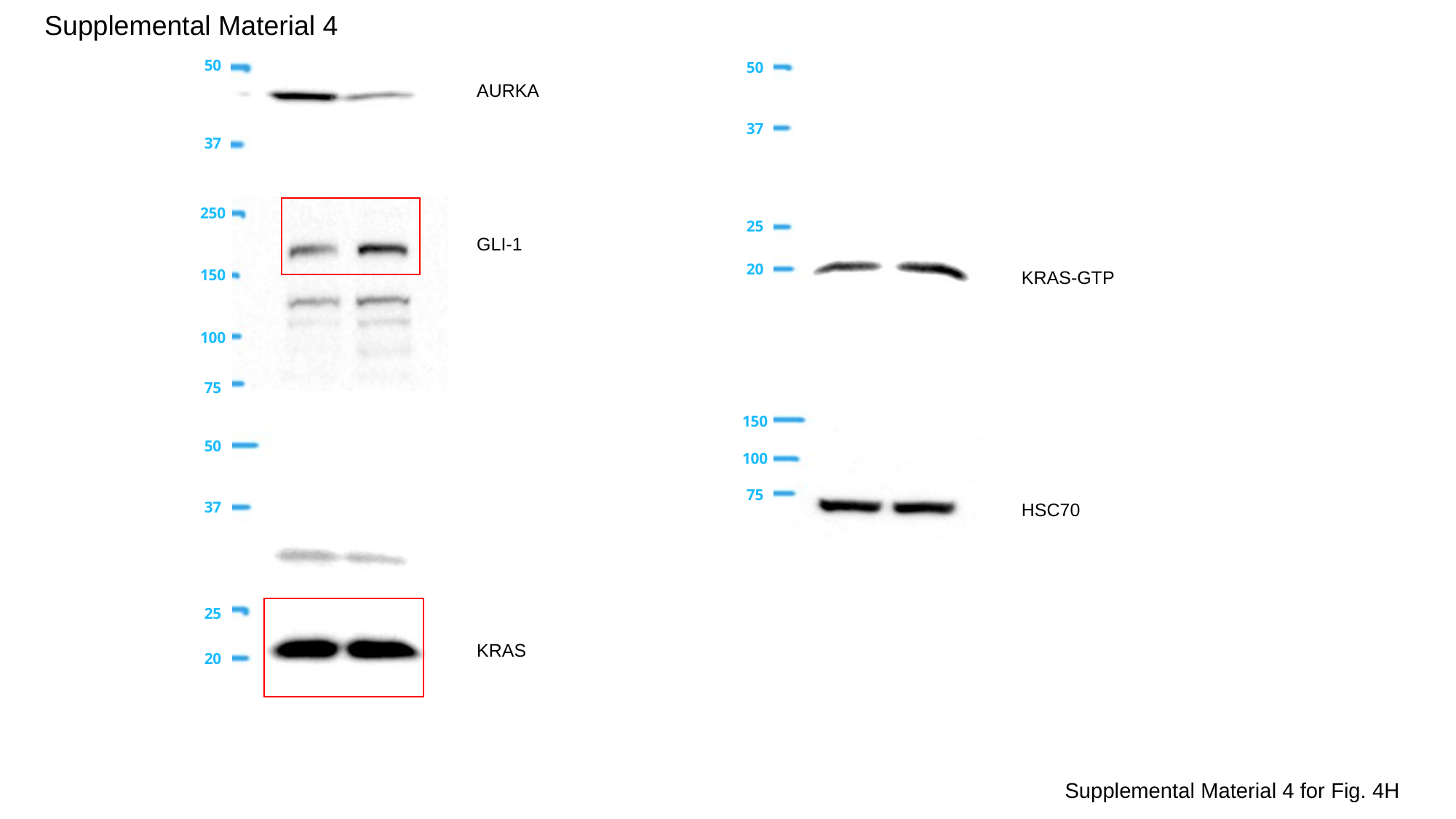

Supplemental Material 4
50
50
AURKA
37
37
250
25
GLI-1
20
150
KRAS-GTP
100
75
150
50
100
75
37
HSC70
25
KRAS
20
Supplemental Material 4 for Fig. 4H

## Slide 12
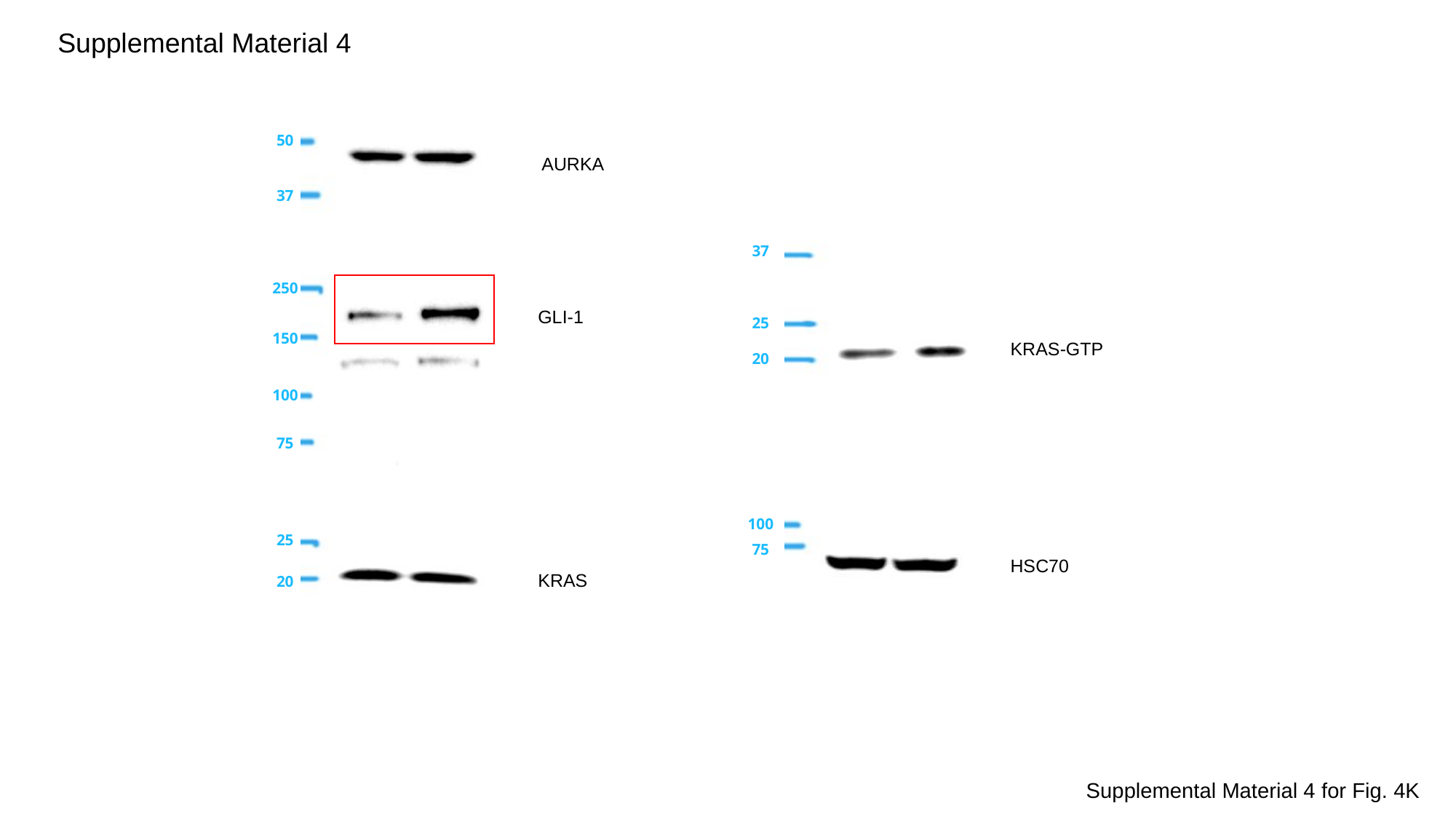

Supplemental Material 4
50
AURKA
37
37
250
GLI-1
25
150
KRAS-GTP
20
100
75
100
25
75
HSC70
KRAS
20
Supplemental Material 4 for Fig. 4K

## Slide 13
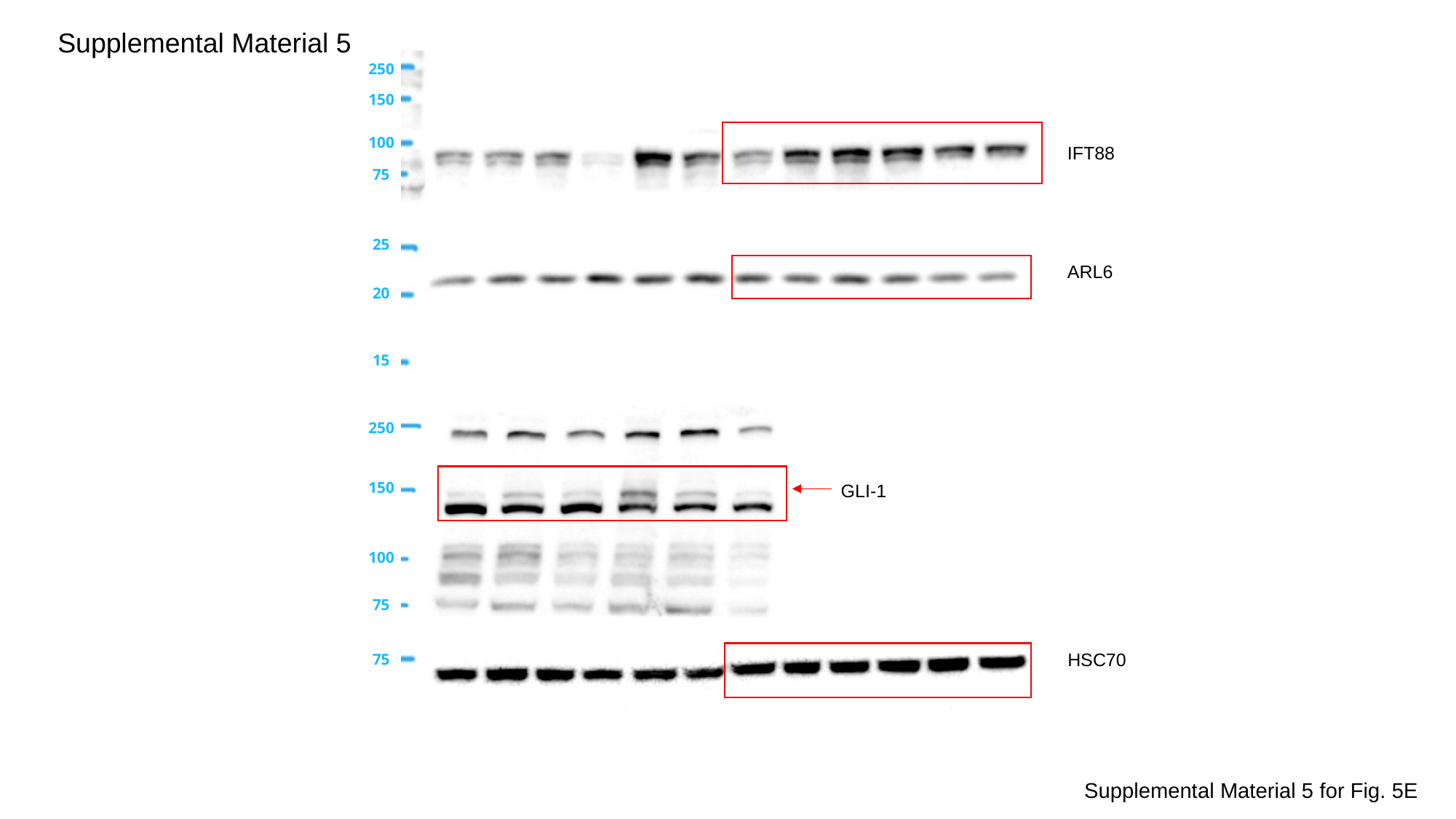

Supplemental Material 5
250
150
100
IFT88
75
25
ARL6
20
15
250
150
GLI-1
100
75
HSC70
75
Supplemental Material 5 for Fig. 5E

## Slide 14
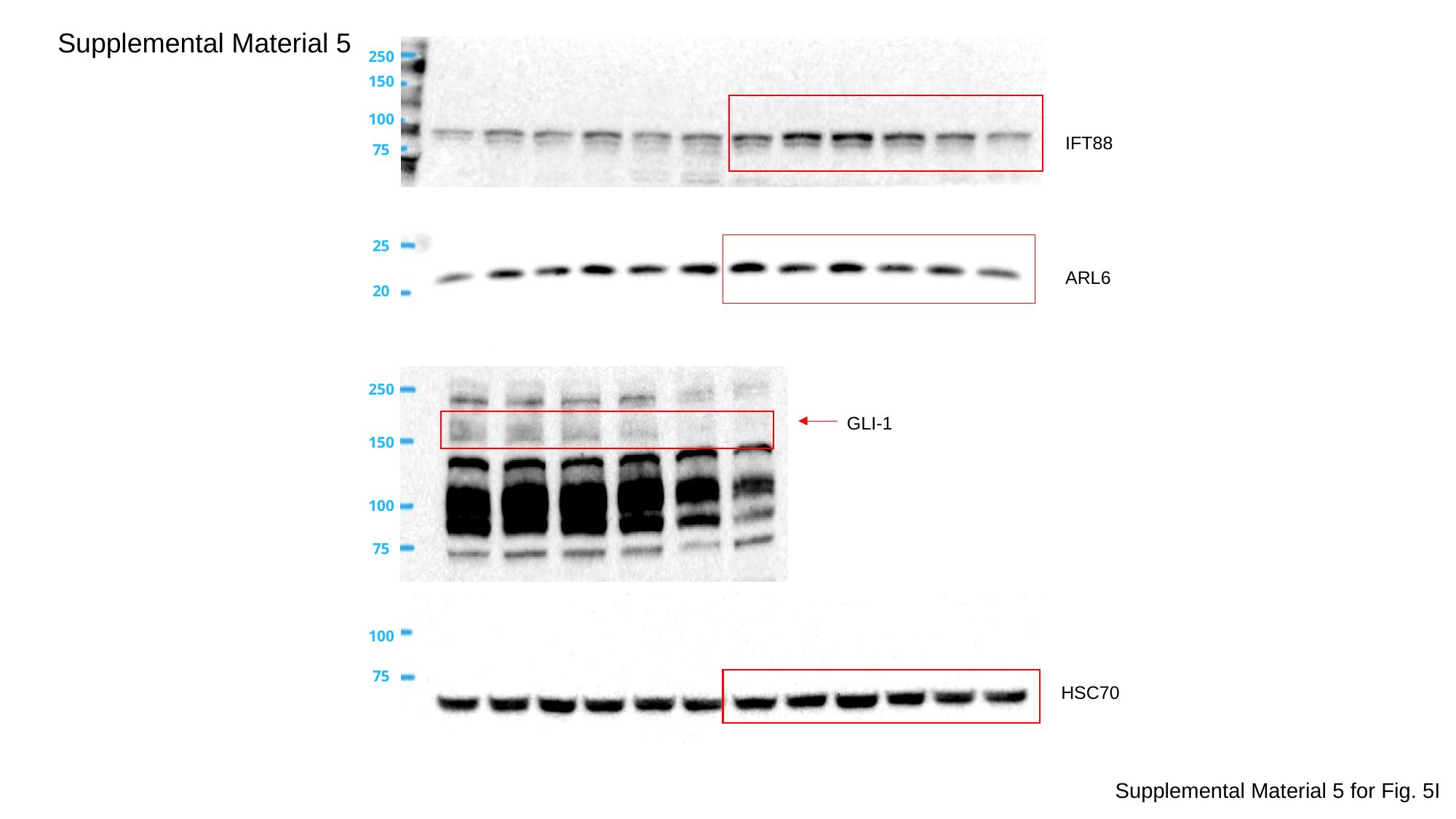

Supplemental Material 5
250
150
100
IFT88
75
25
ARL6
20
250
GLI-1
150
100
75
100
75
HSC70
Supplemental Material 5 for Fig. 5I

## Slide 15
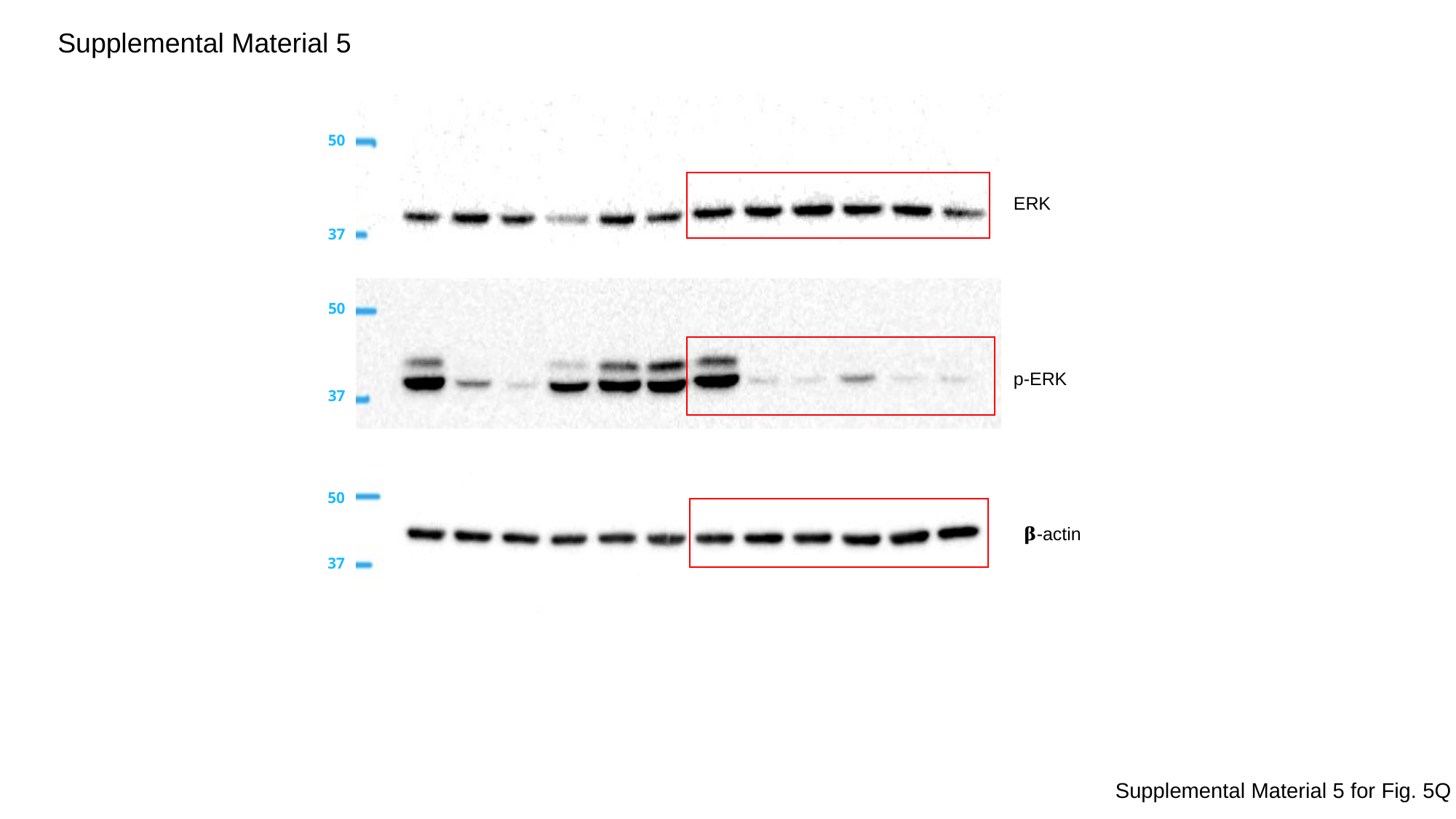

Supplemental Material 5
50
ERK
37
50
p-ERK
37
50
𝛃-actin
37
Supplemental Material 5 for Fig. 5Q

## Slide 16
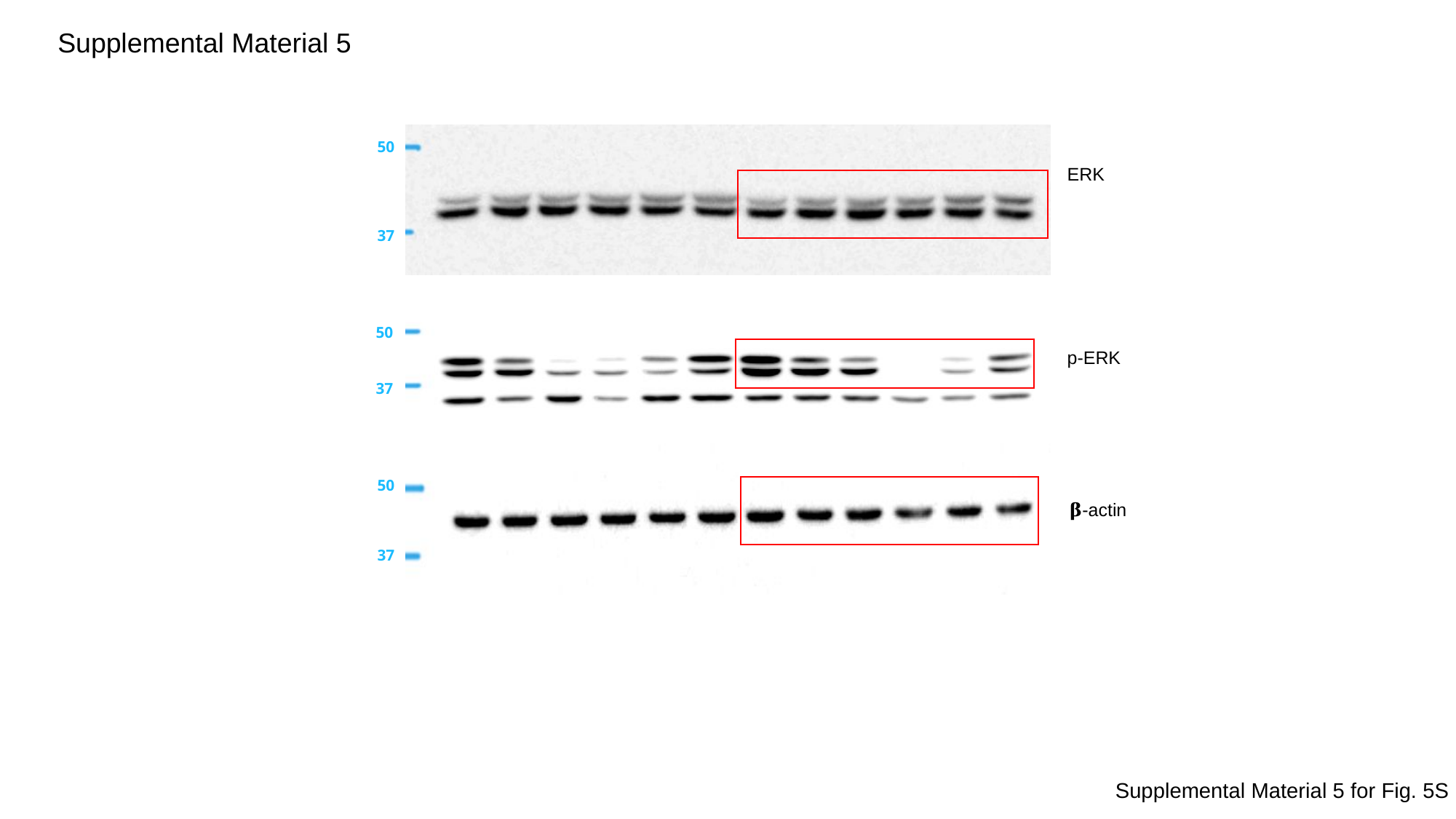

Supplemental Material 5
50
ERK
37
50
p-ERK
37
50
𝛃-actin
37
Supplemental Material 5 for Fig. 5S

## Slide 17
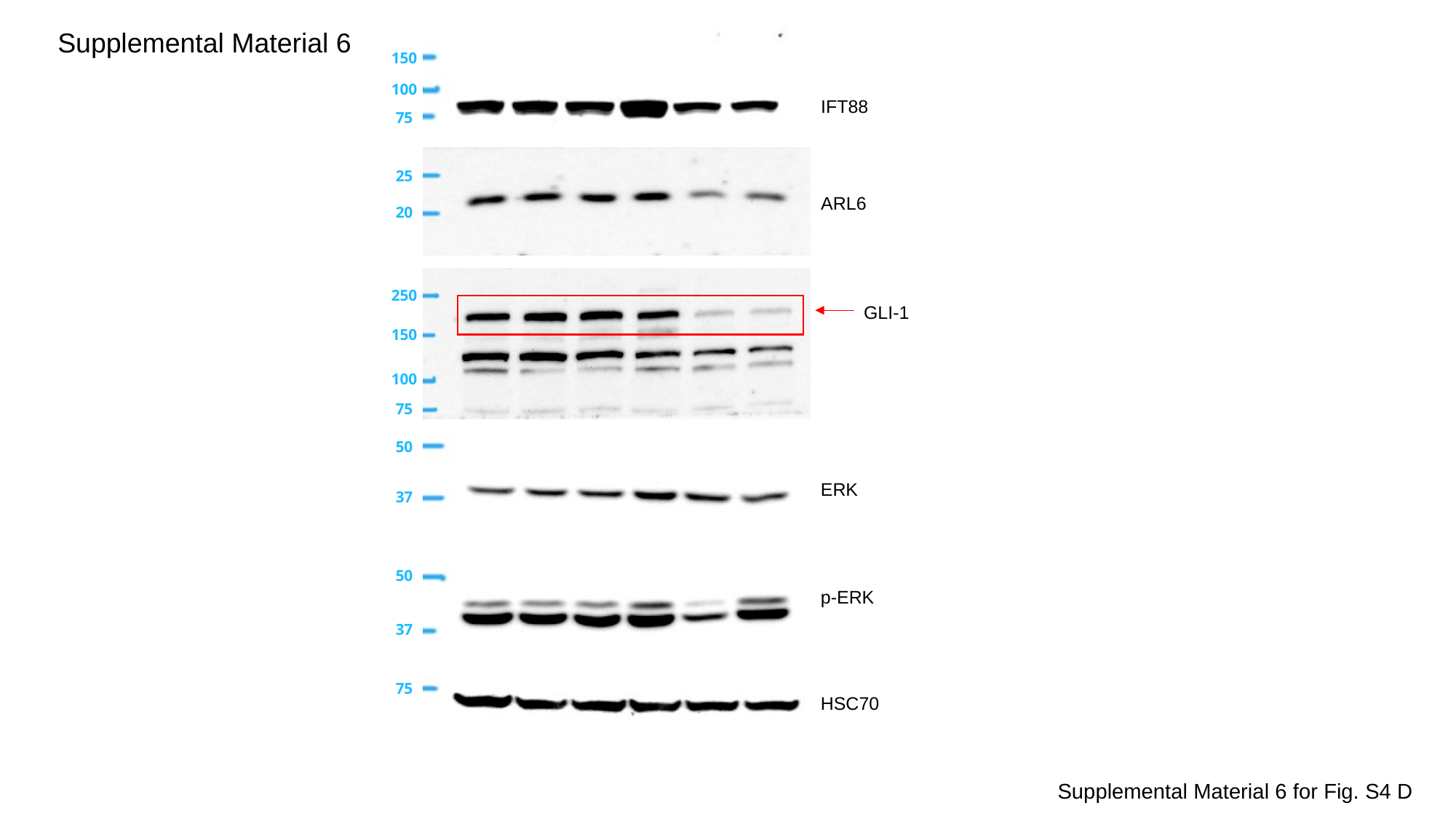

Supplemental Material 6
150
100
IFT88
75
25
ARL6
20
250
GLI-1
150
100
75
50
ERK
37
50
p-ERK
37
75
HSC70
Supplemental Material 6 for Fig. S4 D

## Slide 18
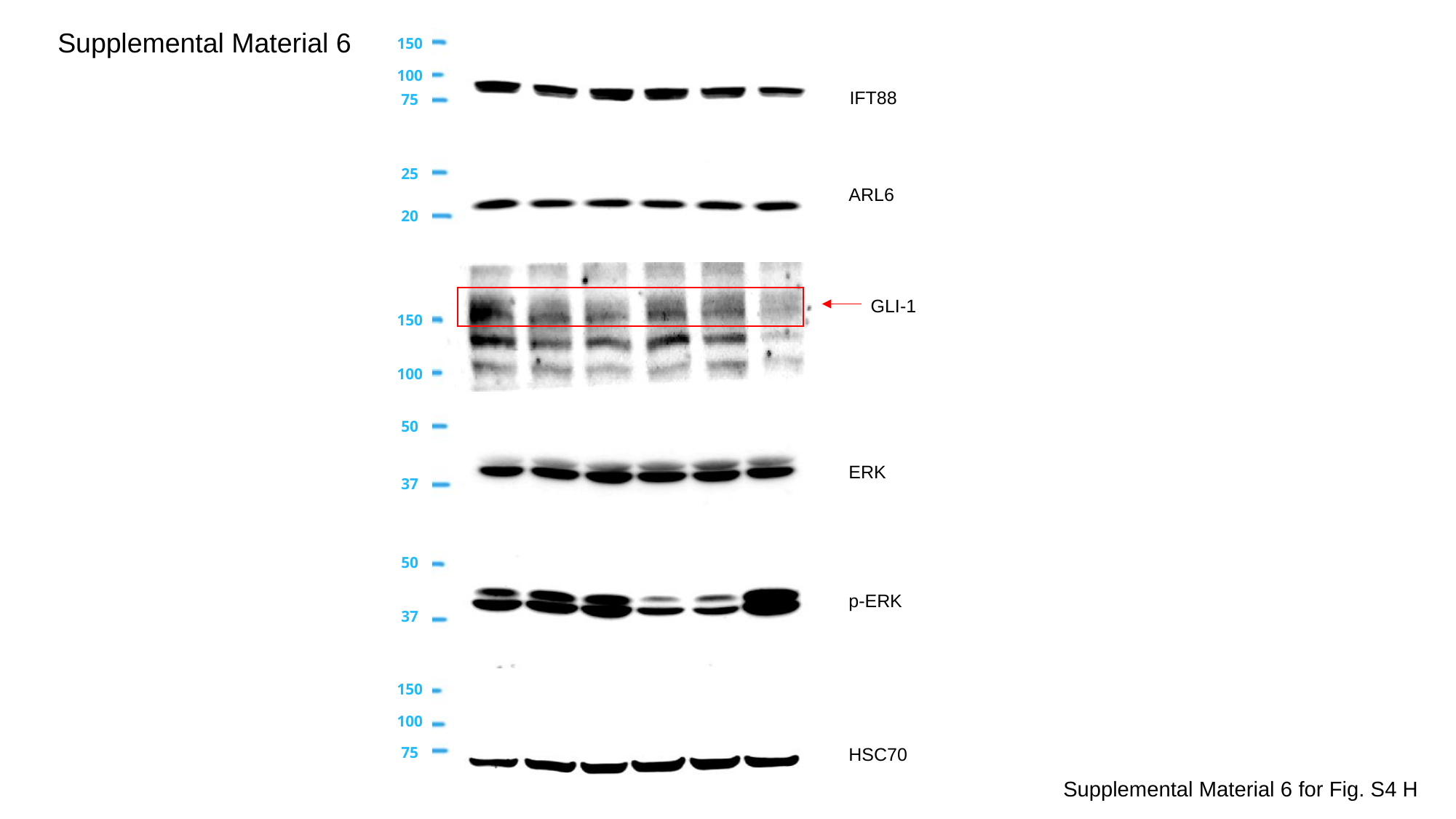

Supplemental Material 6
150
100
IFT88
75
25
ARL6
20
GLI-1
150
100
50
ERK
37
50
p-ERK
37
150
100
75
HSC70
Supplemental Material 6 for Fig. S4 H

## Slide 19
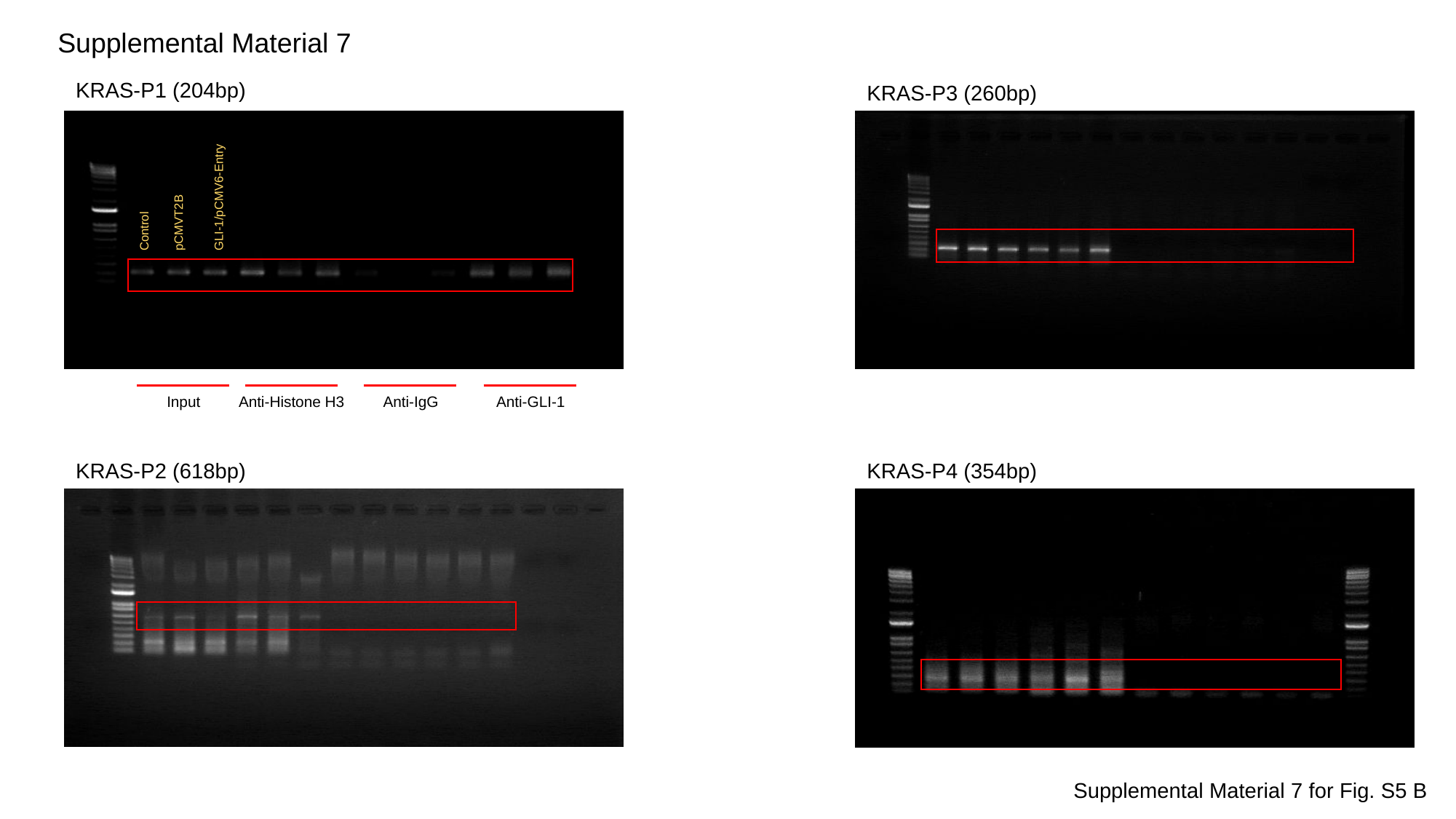

Supplemental Material 7
KRAS-P1 (204bp)
KRAS-P3 (260bp)
GLI-1/pCMV6-Entry
pCMVT2B
Control
Input
Anti-Histone H3
Anti-IgG
Anti-GLI-1
KRAS-P2 (618bp)
KRAS-P4 (354bp)
Supplemental Material 7 for Fig. S5 B

## Slide 20
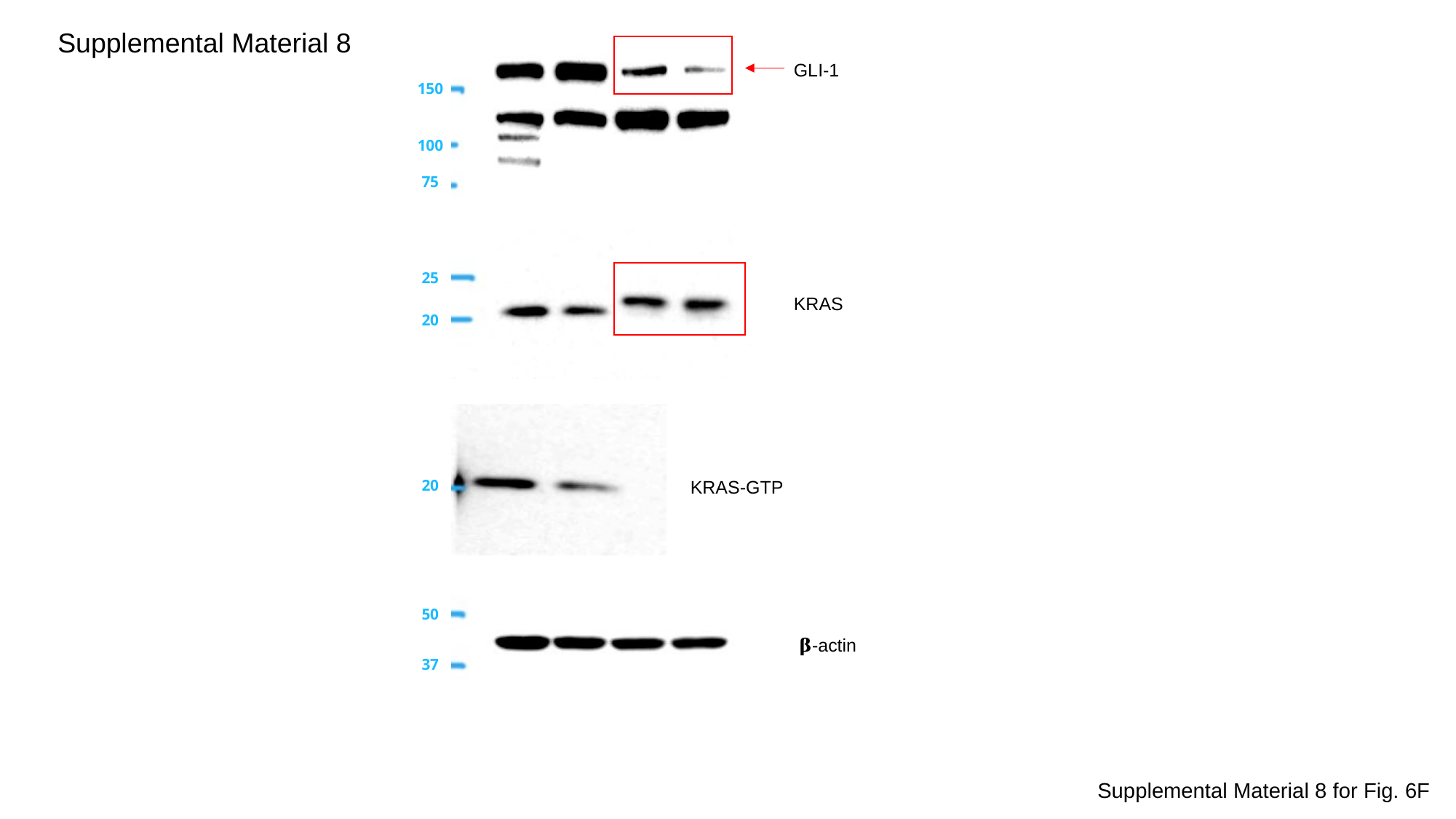

Supplemental Material 8
GLI-1
150
100
75
25
KRAS
20
20
KRAS-GTP
50
𝛃-actin
37
Supplemental Material 8 for Fig. 6F
